# Supplementary material for: Genomic and transcriptomic analyses of aortic stenosis enhance therapeutic target discovery and disease prediction
Source: Nat Genet. 2025 Dec 19;58(1):57–66. doi: 10.1038/s41588-025-02417-6 (PMC12807872; doi:10.1038/s41588-025-02417-6)
Supplement: Supplementary file 1 — Supplementary Methods, Figs. 1–15 and Note (Author funding and Acknowledgements). [file 41588_2025_2417_MOESM1_ESM.pdf]

nature genetics

Supplementary information

https://doi.org/10.1038/s41588-025-02417-6

Genomic and transcriptomic analyses of aortic stenosis enhance therapeutic target discovery and disease prediction

In the format provided by the authors and unedited

Supplementary Material

Table of Contents:

I. **Supplemental Methods**

   a. **Cohort Descriptions**

II. **Supplemental Figures**

   a. **Supplemental Figure 1:** Schematic overview of study design

   b. **Supplemental Figure 2:** Manhattan and QQ plot for multi-ancestry genome-wide association study of aortic stenosis in autosomes

   c. **Supplemental Figure 3:** Manhattan plot of multi-ancestry X-chromosome analysis

   d. **Supplemental Figure 4:** Manhattan and QQ plot for European ancestry genome-wide association study of aortic stenosis in autosomes

   e. **Supplemental Figure 5:** Manhattan and QQ plot for African ancestry genome-wide association study of aortic stenosis in autosomes

   f. **Supplemental Figure 6:** Manhattan and QQ plot for Hispanic ancestry genome-wide association study of aortic stenosis in autosomes

   g. **Supplemental Figure 7:** Manhattan and QQ plot for East Asian ancestry genome-wide association study of aortic stenosis in autosomes

   h. **Supplemental Figure 8:** Manhattan and QQ plot for South Asian ancestry genome-wide association study of aortic stenosis in autosomes

   i. **Supplemental Figure 9:** Deming regression of multi-ancestry lead SNP effect estimates among African, Hispanic, East Asian, and South Asian ancestries.

j. **Supplemental Figure 10:** Manhattan and QQ plot for female sex genome-wide association study of aortic stenosis in autosomes

k. **Supplemental Figure 11:** Manhattan and QQ plot for male sex genome-wide association study of aortic stenosis in autosomes

l. **Supplemental Figure 12:** Similarity matrix of representative ontologies for aortic stenosis

m. **Supplemental Figure 13**: Manhattan plot of phenome-wide association study results for aortic stenosis GWAS lead variants in the Million Veteran Program.

n. **Supplemental Figure 14:** Kaplan Meier curves depicting cumulative diagnosis of aortic stenosis by quintiles of genetic risk in the UKB and TIMI clinical trials.

o. **Supplemental Figure 15:** Forest plot comparing the C-index for individual risk factors to a composite of clinical risk factors or clinical risk factors with genetic risk in the UK Biobank and TIMI clinical trials

III. Author Funding and Acknowledgements

IV. References

Cohort Descriptions

All of Us

All of Us is a United States biobank started in 2018 which collects biospecimens from consenting individuals over 18 years of age with a goal of improving the representation of underrepresented populations[1]. All of Us contains over 175,000 participants. Genotyping was performed using short read whole genome sequencing. Aortic stenosis case/control status was determined using the International Aortic Valve Genetics Consortium's provided definitions. A GWAS for aortic stenosis was performed in autosomes using imputed data from 555 European genetic ancestry cases and 47,113 European genetic ancestry controls. The associations were modeled using logistic regression in REGENIE with adjustment for age, age^2, sex (except sex-stratified analyses), and ancestry-specific principal components.

Biobank Japan

The BioBank Japan (BBJ) is a hospital-based national biobank project that collects DNA, serum samples and clinical information[2]. BBJ contains approximately 200,000 participants recruited from cooperative medical institutes. Genotyping was performed using the Illumina HumanOmniExpress Genotyping BeadChip or a combination of Illumina HumanOmniExpress and HumanExome BeadChips, and following standard genotyping quality control, the genotyped data were imputed using BBJ1K WGS (n = 1037) + 1KG phase3v5 (n = 2504). Aortic stenosis case/control status was determined using the International Aortic Valve Genetics Consortium's provided definitions. A GWAS for aortic stenosis was performed in autosomes using imputed data from 1,403 East Asian genetic ancestry cases and 130,229 East Asian genetic ancestry controls.

Additionally, GWAS were performed for autosomes and chromosome X in sex-stratified data (746 male cases, 71,596 male controls; 657 female cases, 58,633 female controls). The associations were modeled using mixed model logistic regression in SAIGE (version 0.42.1) with adjustment for age, age^2, sex (except sex-stratified analyses), and 10 ancestry-specific principal components.

BioMe

The Mount Sinai Bio*Me* Biobank is an ongoing electronic health record-linked biorepository that enrolls participants non-selectively from the Mount Sinai Health System, comprising approximately 60,000 participants[3]. Genotyping was performed using the Global Screen Array (GSA), for ~40,000 samples, and the Global Diversity Array (GDA), for ~20,000 samples and following standard genotyping quality control. The genotyped data were imputed using the TOPMed imputation server (version r2). Aortic stenosis case/control status was determined using the International Aortic Valve Genetics Consortium's provided definitions. A GWAS for aortic stenosis was performed in autosomes separately by genetic ancestry using imputed data from 245 European genetic ancestry cases, 14,449 European genetic ancestry controls, 188 Hispanic genetic ancestry case, and 8,118 Hispanic genetic ancestry controls. Additionally, GWAS was performed for autosomes and the X chromosome in sex-stratified data. The associations were modelled using logistic regression in REGENIE with adjustment for age, age^2, sex, (except sex stratified analyses), genotyping chip and ancestry specific principal components.

BioVU

BioVU (Vanderbilt University) is Vanderbilt's biorepository of DNA and genetic data extracted from discarded blood collected during routine clinical testing and linked to de-identified medical records[4]. BioVU contains approximately 318,000 participants. Genotyping was performed using the Infinium Expanded Multi-Ethnic Genotyping Array ($MEGA^{EX}$), and following standard genotyping quality control, the genotyped data were imputed using the TOPMed imputation server (version r2). Aortic stenosis case/control status was determined using the International Aortic Valve Genetics Consortium's provided definitions. A GWAS for aortic stenosis was performed in autosomes separately by genetic ancestry using imputed data from 2,938 European genetic ancestry cases, 70,915 European genetic ancestry controls, 259 African genetic ancestry cases, and 15,595 African genetic ancestry controls. Additionally, GWAS were performed for autosomes in sex stratified data. The associations were modelled using logistic regression in SAIGE with adjustment for age, age^2, sex (except sex-stratified analyses), and ancestry-specific principal components.

CATHGEN

The CATHeterization GENetics (CATHGEN) cohort contains 9,334 participants consecutively referred to the Duke University Hospital cardiac catheterization between 2001 and 2010[5]. Genotyping was performed using the Illumina Human Omni1-Quad Infinium Bead Chip, and following standard genotyping quality control, the genotyped data were imputed using the TOPMed imputation server. Aortic stenosis case/control status was determined using the International Aortic Valve Genetics Consortium's provided definitions. A GWAS for aortic stenosis was performed in autosomes using imputed data from 202 European genetic ancestry cases and

1,685 European genetic ancestry controls. Additionally, GWAS were performed for autosomes in sex-stratified data (108 male cases, 1,036 male controls). The associations were modeled using logistic regression in REGENIE with adjustment for age, age^2, sex (except sex-stratified analyses), and ancestry-specific principal components.

Cartagene

Cartagene (CaG) is a Quebec population-based cohort and a prospective study of men and women aged between 40-69 years. CaG contains approximately 42,000 participants recruited randomly from the Quebec population. Array-based genotyping was performed on approximately 30,000 with standard genotyping quality control, and the genotyped data were imputed using the TOPMed imputation server. Aortic stenosis case/control status was determined using the International Aortic Valve Genetics Consortium's provided definitions. The GWAS for aortic stenosis (197 cases and 5129 controls) was performed for autosomes in individuals of European genetic. Additionally, GWAS were performed for autosomes and the X Chromosome in males (132 cases and 2403 controls). The associations were modeled using logistic regression in REGENIE with adjustment for age, age^2, sex (except sex-stratified analyses), and five ancestry-specific principal components.

CAVS France

CAVS-France contains 8,130 participants recruited between the years 2001 and 2017, inclusively. 1,663 severe aortic stenosis cases confirmed via echocardiography were recruited from the Angers, Rennes, and Nantes University Hospitals as part of a biobank operated by l'institut du

thorax in Nantes, France[6]. Separate recruitment by the Bichat University Hospital enrolled 1,500 echocardiography-confirmed cases of aortic stenosis as part of the COFRASA-GENERAC study[7]. Controls were drawn from the DESIR[8] and PREGO[3] cohorts, which recruited individuals from western France. Genotyping was performed using on one hand the Axiom™ Genome-Wide CEU Array (2,046 participants) and on the other hand the Axiom™ Precision Medicine Research (PMRA) array (6,084 participants), and following standard genotyping quality control, the genotyped data were imputed using the TOPMed imputation server. Aortic stenosis case/control status was determined using the International Aortic Valve Genetics Consortium's provided definitions. GWAS for aortic stenosis were performed in each cohort in autosomes using imputed data from 1,751 European genetic ancestry cases and 6,379 European genetic ancestry controls. Additionally, GWAS were performed for autosomes in sex stratified data (1,072 male cases, 3,501 male controls; 679 female cases, 2,878 female controls). The associations were modeled using logistic regression in SAIGEv0.42 with adjustment for age centered per state[2], sex (except sex-stratified analyses), and ancestry-specific principal components. Analysis was performed separately for individuals genotyped on the CEU array and PMRA array and meta-analyzed using inverse variance weighted meta-analysis.

Colorado Center for Precision Medicine

The Colorado Center for Precision Medicine (CCPM) is a biobank developed by the University of Colorado Anschutz Medical Campus and UCHealth which currently has more than 200,000 participants and over 33,000 genotyped individuals[9]. Genotyping was performed using the Illumina MEGA/exome array and following standard genotyping quality control, the genotyped

data were imputed using TOPMed (version r2). Aortic stenosis case/control status was determined using the International Aortic Valve Genetics Consortium's provided definitions. A GWAS for aortic stenosis was performed in autosomes using imputed data from 1,137 European genetic ancestry cases and 53,105 European genetic ancestry controls. Additionally, GWAS were performed for autosomes in sex-stratified data (642 male cases, 20,332 male controls; 495 female cases, 32,773 female controls). The associations were modeled using logistic regression in REGENIE (v3.2.1) with adjustment for age, age^2, sex (except sex-stratified analyses), and ancestry-specific principal components.

The Cohort of Swedish Men

We used data from the Swedish Infrastructure for Medical Population-Based Life-Course and Environmental Research (SIMPLER; https://www.simpler4health.se/), which consists of two large population-based cohorts: the Swedish Mammography Cohort (SMC) and the Cohort of Swedish Men (COSM). The Cohort of Swedish Men (COSM) was established in 1997 when ~50,000 men born between 1918 and 1952 and residing in Västmanland and Örebro counties completed a questionnaire[10-13]. In a clinical subcohort (COSMC), around 7384 participants of the COSM and SMC studies who lived in Västerås (a city in Västmanland County) participated in a health examination between 2010 and 2019, and they provided over-night fasting blood samples. Samples were genotyped using Illumina Infinium Global Screening Array version 3 (GSAv3), and following standard genotyping quality control, the genotyped data were imputed by use of chr1-22,X: Michigan Imputation Server v1.2.4 using Eagle v2.4 + minimac v4 and both 1000G phase3 (v5) in tgp.ph3/ and HRC v1.1 in hrc1.1/ as reference panels. Aortic stenosis case/control status

was determined using the International Aortic Valve Genetics Consortium's provided definitions. A GWAS for aortic stenosis was performed in autosomes using imputed data from 198 European genetic ancestry cases and 6,152 European genetic ancestry controls. Additionally, in sex-stratified data, GWAS was performed for autosomes and the X-chromosome (157 male cases, 3,958 male controls). The associations were modeled using logistic regression with adjustment for age, sex (except sex-stratified analyses), and ancestry-specific principal components.

Copenhagen Hospital Biobank and the Danish Blood Donor Study

The Copenhagen Hospital Biobank (CHB) was started in 2009 and is a collection of surplus material from diagnostic testing on patients admitted to general hospitals in the Capital region of Copenhagen[14]. The current analysis was done under the CHB Cardiovascular Disease Cohort (CHB-CVDC) (approval number: NVK-1708829, P-2019-93) [15]. The analysis was combined with data on participants in The Danish Blood Donor Study (DBDS) (approval number: NVK-1700407, P-2019-99) [16]. DBDS has since 2010 included blood donors into a prospective cohort study and biobank. Genotyping of both CHB-CVDC and DBDS was performed at deCODE Genetics using the Infinium Global Screening Array (Illumina) and following standard genotyping quality control, the genotyped data were imputed using the North European reference panel at deCODE Genetics. Aortic stenosis case/control status was determined using the International Aortic Valve Genetics Consortium's provided definitions. A GWAS for aortic stenosis was performed in autosomes using imputed data from 9,654 European genetic ancestry cases and 226,682 European genetic ancestry controls. Additionally, GWAS were performed for autosomes and chromosome X in sex stratified data (5,665 male cases, 116,555 male controls; 3,989 female cases, 110,127 female

controls). The associations were modeled using logistic regression in REGENIE with adjustment for age, age^2, sex (except sex-stratified analyses), and ancestry-specific principal components.

deCODE

The deCODE dataset included Icelandic patients diagnosed with CAVS in the years 1983-2019 at Landspitali – The National University Hospital in Reykjavik, the only tertiary referral center in Iceland. Following long-range phasing, variants identified in 63,460 whole genome sequenced (WGS) Icelanders were imputed into 173,025 individuals chip-genotyped employing multiple Illumina platforms[17]. Familial imputation of genotypes in first- and second-degree relatives was used to increase sample size[17]. Aortic stenosis case/control status was determined using the International Aortic Valve Genetics Consortium's provided definitions. A GWAS for aortic stenosis was performed in autosomes using imputed data from 3,133 Icelandic cases and 286,089 Icelandic controls. Additionally, GWAS were performed for autosomes and chromosome X in sex stratified data (1,640 male cases, 145,105 male controls; 1,493 female cases, 103,884 female controls). The associations were tested using logistic regression under the additive model, including current age or age at death, sex (except sex-stratified analyses), and county of birth as covariates. For the association analyses, we used software developed at deCODE genetics[17]. We used LD score regression intercepts[18] to adjust the $\chi 2$ statistics and avoid inflation due cryptic relatedness and stratification, using a set of 1.1 million variants. P values were calculated from the adjusted $\chi 2$ results.

Estonian Biobank

The Estonian Biobank is a volunteer-based sample of adult Estonians recruited by general practitioners and medical personnel throughout the country[19]. Genotyping was performed using the Global Screening Array v3.0_EST, and following standard genotyping quality control, the genotyped data were imputed using a population specific reference panel consisting of 2,695 whole genome sequenced samples. Aortic stenosis case/control status was determined using the International Aortic Valve Genetics Consortium's provided definitions. A GWAS for aortic stenosis was performed in autosomes using imputed data from 1,444 European genetic ancestry cases and 202,790 European genetic ancestry controls. Additionally, GWAS were performed for autosomes and chromosome X in sex stratified data (719 male cases, 70,533 male controls; 1,188 female cases, 134,191 female controls). The associations were modeled using logistic regression in REGENIE (ver 3.0.3) with adjustment for age, age2, sex (except sex-stratified analyses), and ancestry-specific principal components.

FinnGen

The FinnGen Study is an ongoing prospective cohort study that combined population-based legacy cohorts, disease-based cohorts, and individuals recruited by biobanks[20]. FinnGen contains approximately 500,000 participants recruited in university hospital settings. Genotyping was performed using the custom Axion FinnGen1 array, as well as several non-custom legacy chips, and following standard genotyping quality control, the genotyped data were imputed using the population-specific Sequencing Initiative Suomi (SISu) v.3 imputation reference panel. Aortic stenosis case/control status was determined using the International Aortic Valve Genetics Consortium's provided definitions. Since FinnGen records procedure codes using NOMESCO

instead of CPT4, the CPT4 codes for aortic valve replacement were replaced with the comparable NOMESCO codes FMA (repair of aortic valve for stenosis) and FMB (expansion of aortic ostium). A GWAS for aortic stenosis was performed in autosomes using imputed data from 8,049 European genetic ancestry cases and 439,193 European genetic ancestry controls. Additionally, GWAS were performed for autosomes and chromosome X in sex stratified data (5,020 male cases, 190,609 male controls; 3,029 female cases, 248,584 female controls). The associations were modelled using logistic regression in REGENIE with adjustment for age, age^2, sex (except sex stratified analyses), and ancestry specific principal components.

GERA

The Genetic Epidemiology Research on Adult Health and Aging (GERA) cohort is composed of over 100,000 ethnically diverse members of the Kaiser Permanente Medical Care Plan, Northern California Region who are also participants of the Kaiser Permanente Research Program on Genes, Environment, and Health[21]. Genome-wide genotyping was performed on ethnicity-specific Affymetrix Axiom arrays, with the European-ancestry participants genotyped on the Affymetrix Axiom Genome-Wide EUR array. Following standard genotyping quality control, the genotyped data were imputed using the TOPmed imputation server. Aortic stenosis case/control status was determined using the International Aortic Valve Genetics Consortium's provided definitions. A GWAS for aortic stenosis was performed in autosomes using imputed data from 3,416 European genetic ancestry cases and 51,070 European genetic ancestry controls. Additionally, GWAS were performed for autosomes and chromosome X in sex-stratified data (1909 male cases, 21460 male controls; 1507 female cases, 29610 female controls). The

associations were modeled using logistic regression in REGENIE with adjustment for age, age^2, sex (except sex-stratified analyses), and 10 ancestry-specific principal components.

German Aortic Stenosis Cohort

The German Aortic Stenosis Cohort recruited patients from the German Heart Center Munich and from the University of Bonn/Marburg and contains approximately 5732 participants with aortic valve stenosis recruited. Diagnosis of aortic valve stenosis was based on echocardiography defined as thickening of the aortic valve and evidence of an increased peak velocity of >2.5 m/s using cw-doppler. Furthermore, patients with a history of aortic valve replacement for severe aortic valve stenosis were recruited. Genotyping in the German aortic stenosis cohort was performed using the Illumina Global Screening Array (GSA) v3.0, and following standard genotyping quality control, the genotyped data were imputed using the TOPMed imputation server. Controls for the German GWAS were part of the Heinz-Nixdorf Recall (HNR) study, the PROCAM-2 Study, the PopGen Biobank and the FOCUS study. In the controls cohorts all participants with a history of any valve replacement were excluded. Aortic stenosis case/control status was determined using the International Aortic Valve Genetics Consortium's provided definitions. A GWAS for aortic stenosis was performed in autosomes using imputed data from 5,732 European genetic ancestry cases and 15,367 European genetic ancestry controls. The associations were modeled using Firth logistic regression as implemented in plink2 with adjustment for age and sex and ancestry-specific principal components

Genes & Health

Genes & Health is a UK based cohort of Pakistani and Bangladeshi individuals recruited in East London[22]. Genotyping was performed using the Illumina Infinium Global Screening Array, and following standard genotyping quality control, the genotyped data were imputed using TOPMed (version r2). Aortic stenosis case/control status was determined using the International Aortic Valve Genetics Consortium's provided definitions. A GWAS for aortic stenosis was performed in autosomes using imputed data from 109 SAS genetic ancestry cases and 43,841 SAS genetic ancestry controls. The associations were modeled using logistic regression in Regenie with adjustment for age, age^2, sex (except sex-stratified analyses), and ancestry-specific principal components.

HUNT

The Trøndelag Health Study (HUNT) is a large, population-based health study based in Trøndelag County, Norway which has to date recruited over 229,000 adults among whom over 88,000 have had genome-wide genotyping[23]. Genotyping was performed using four illumine arrays (HumanCoreExome12 v1.0, HumanCoreExome12 v1.1, UM HUNT Biobank v1.0, UM HUNT Biobank v2.0) and following standard genotyping quality control, the genotyped data were imputed from the TOPMed reference panel (version r2). Aortic stenosis case/control status was determined using the International Aortic Valve Genetics Consortium's provided definitions. A GWAS for aortic stenosis was performed in autosomes using imputed data from 2,202 European genetic ancestry cases and 65,864 European genetic ancestry controls. Additionally, GWAS were performed for autosomes and chromosome X in sex stratified data (1,055 male cases, 30,858 male controls; 1,147 female cases, 35,006 female controls). The associations were modeled using

logistic regression in SAIGE v.1.0.3 with adjustment for age, age^2, sex (except sex-stratified analyses), and the first 10 ancestry-specific principal components.

HerediGene (Intermountain)

The HerediGene Population Study is a collaboration between deCODE genetics and Intermountain Health in Utah which recruits individuals in the Intermountain Health system, encompassing 22 hospitals in the Mountain West region of Utah and Idaho[24]. Sequence variants, identified through WGS of 23,288 Americans of European ancestry from Utah, were imputed into 138,006 individuals enrolled at multiple Intermountain Healthcare facilities and chip-typed at deCODE genetics using Illumina platform. The imputation was based on long-range phasing and sequence variant calling that was performed jointly[25] for several sample-sets (Danish, N-American, Iranian, Swedish, and Dutch), for which 50,839 have been WGS and 1,041,174 chip-typed. The associations were tested using logistic regression under the additive model, including current age or age at death, sex (except sex-stratified analyses), and ancestry-specific principal components as covariates. For the association analyses, we used software developed at deCODE genetics[17]. We used LD score regression intercepts[18] to adjust the $\chi2$ statistics and avoid inflation due cryptic relatedness and stratification, using a set of 1.1 million variants. P values were calculated from the adjusted $\chi2$ results. Aortic stenosis case/control status was determined using the International Aortic Valve Genetics Consortium's provided definitions. A GWAS for aortic stenosis was performed in autosomes using imputed data from 3,627 European genetic ancestry cases and 65,482 European genetic ancestry controls. Additionally, GWAS were performed for autosomes and chromosome X in sex stratified data (2,130 male cases, 27,669 male controls;

1,497 female cases, 37,773 female controls). Participants in the HerediGene population study are voluntary US residents over the age of 18 years, who gave permission to link anonymized genotypic data with electronic health records.

Leicester

Cases from Leicester (part of the GeneCast aortic stenosis study) were recruited at the University of Leicester in the UK. Individuals with AS were identified by echocardiography (using hemodynamic criteria of a peak velocity > 2.5 m/s or 2.0 m/s among individuals with severe left ventricular systolic dysfunction). Genotyping was performed using the UK Biobank Axiom WCS, and following standard genotyping quality control, the genotyped data were imputed using the TOPMed imputation server (version r2). A GWAS for aortic stenosis was performed in autosomes using imputed data from 1,927 European genetic ancestry cases and 9,543 European genetic ancestry controls. The associations were modeled using logistic regression with adjustment for age, age^2, sex (except sex-stratified analyses), and ancestry-specific principal components.

Malmo Diet and Cancer

The Malmö Diet and Cancer Study (MDCS) is a community-based prospective cohort of middle-aged individuals from Southern Sweden which included 30 447 participants at a baseline exam in 1991-1996[26]. At baseline, participants filled out a questionnaire, underwent anthropometric measurements and donated peripheral venous blood samples from which DNA was isolated for GWAS genotyping. Genotyping was performed using the Illumina Infinium Global Screening Array v1, and following standard genotyping quality control, the genotyped data were imputed using

the Michigan Imputation Server. Aortic stenosis case/control status was determined from nation-wide hospital registers with high validity as described previously and in accordance with the International Aortic Valve Genetics Consortium's provided definitions. A GWAS for aortic stenosis was performed in autosomes using imputed data from 1,003 cases of European genetic ancestry cases and 27,908 controls of European genetic ancestry. Additionally, GWAS were performed for autosomes and chromosome X in sex stratified data (477 male cases, 10,981 male controls; 526 female cases, 16,927 female controls). The associations were modelled using logistic regression in REGENIE with adjustment for age, age^2, sex (except sex stratified analyses), and ancestry specific principal components. The study was approved by the local ethics committee and all participants provided written informed consent.

Mass General Brigham Biobank

The Mass General Brigham Biobank (MGBB) represents an academic biobank affiliated with the Mass General Brigham healthcare system, which is the largest healthcare system in the state of Massachusetts[27]. The MGBB has consented over 140,000 individuals to date. Genotyping was performed using the Illumina Global Screening Array and following standard genotyping quality control, the genotyped data were imputed using the TOPMed imputation server (version r2). Aortic stenosis case/control status was determined using the International Aortic Valve Genetics Consortium's provided definitions. A GWAS for aortic stenosis was performed in autosomes using imputed data from 2,368 European genetic ancestry cases and 45,323 European genetic ancestry controls. Additionally, GWAS were performed for autosomes and chromosome X in sex stratified data (1,427 male cases, 19,294 male controls; 941 female cases, 26,028 female controls). The

associations were modeled using logistic regression in SAIGE with adjustment for age, age^2, sex (except sex-stratified analyses), and ancestry-specific principal components.

Million Veteran Program

The Million Veteran Program is an observational cohort study and large biobank in the Department of Veterans Affairs VA Healthcare System, with enrollment begun in 2011[28]. Genotyping was performed using a custom Axiom array (MVP1.0), and following standard genotyping quality control, the genotyped data were imputed using the TOPMed imputation server (version r2). Aortic stenosis case/control status was determined using the International Aortic Valve Genetics Consortium's provided definitions. A GWAS for aortic stenosis was performed in autosomes separately by genetic ancestry using imputed data from 22,235 European genetic ancestry cases, 421,730 European genetic ancestry controls, 1,215 Hispanic genetic ancestry cases, and 48,326 Hispanic genetic ancestry controls, 2,531 African genetic ancestry cases and 115,520 African ancestry controls. Additionally, GWAS were performed for autosomes and the X chromosome in sex stratified data. The associations were modeled using logistic regression in REGENIE with adjustment for age, age^2, sex (except sex-stratified analyses), and ancestry-specific principal components.

Penn Medicine Biobank

The Penn Medicine Biobank is an academic biobank at the University of Pennsylvania healthcare system[29]. Genotyping was performed using the Illumina Global Screening Array v.2.0, and following standard genotyping quality control, the genotyped data were imputed using the

TOPMed imputation server (version r2). Aortic stenosis case/control status was determined using the International Aortic Valve Genetics Consortium's provided definitions. A GWAS for aortic stenosis was performed in autosomes using imputed data from 2,507 European genetic ancestry cases and 23,991 European genetic ancestry controls as well as 336 African ancestry cases and 9,909 African ancestry controls. Additionally, GWAS were performed for autosomes and the X chromosome in sex stratified data. The associations were modeled using logistic regression in SAIGE with adjustment for age, age^2, sex (except sex-stratified analyses), and ancestry-specific principal components.

United Kingdom Biobank

The UK Biobank is a prospective cohort of more than 500,000 individuals living in the United Kingdom who were 37-73 years of age at recruitment (2006-2010) [30]. Genotyping was performed on 487,409 samples using the Affymetrix UK Biobank Axiom Array, and following standard genotyping quality control, imputation of the genotyped data was performed using the Haplotype Reference Consortium version r1.1, the 1000 Genomes Project phase 3[31], and the UK10K[32] as reference panels. Aortic stenosis case/control status was determined using the International Aortic Valve Genetics Consortium's provided definitions. A GWAS for aortic stenosis was performed in autosomes using imputed data from 4340 European genetic ancestry cases and 2,622,531 European genetic ancestry controls. Additionally, GWAS were performed for autosomes in sex-stratified data (2765 male cases, 120722 male controls; 1575 female cases, 141809 female controls). The associations were modelled using logistic regression in REGENIE

with adjustment for age, age^2, sex (except sex stratified analyses), genotype batch, and 10 ancestry specific principal components.

The Northern Sweden Health and Disease Study

The Northern Sweden Health and Disease Study (NSHDS) comprises three sub-surveys, the Västerbotten Intervention Program (VIP), the Northerns Sweden MONICA study and the Mammography Screening Program (MSP), whereof VIP and MONICA are actively recruiting. Until 31st December 2014, 140,414 unique subjects of European-ancestry had participated whereof 873 had undergone surgery for disease of the ascending aorta and/or valvular heart disease[33,34]. They were matched on age, sex, type and date of survey, and area of residency via a 1:4 scheme to controls selected from participants in NSHDS without any surgery for disease of the ascending aorta and/or valvular heart disease. Genotyping was performed for 1,853 participants using the Affymetrix UK Biobank Axiom Array r3, yielding 1,699 participants and 760,637 variants which passed standard genotyping quality control. We imputed the genotypes using the TOPMED Imputation Server. A GWAS for aortic stenosis was performed in autosomes using imputed data from 216 European genetic ancestry cases and 428 European genetic ancestry controls. Additionally, GWAS were performed for autosomes in sex stratified data (100 male cases, 200 male controls; 116 female cases, 228 female controls). The associations were modelled using logistic regression in REGENIE with adjustment for age, age^2, sex (except sex stratified analyses), and 5 ancestry specific principal components.

The Swedish Mammography Cohort

We used data from the Swedish Infrastructure for Medical Population-Based Life-Course and Environmental Research (SIMPLER; https://www.simpler4health.se/), which consists of two large population-based cohorts: the Swedish Mammography Cohort (SMC) and the Cohort of Swedish Men (COSM). The Swedish Mammography Cohort (SMC) is a population-based cohort study established in 1987-1990 when ~60,000 women who were born between 1914 and 1948 and living in Uppsala and Västmanland countries agreed to participate in the study and in a mammography screening program[10-13]. Participants have completed questionnaires in 1987-1990, 1997, 2008, 2009, and 2019. In a clinical subcohort (SMCC), 5022 SMC participants who resided in Uppsala provided a blood sample between 2003 and 2009. Samples were genotyped using the Illumina Infinium Global Screening Array Multiple Disease version 1 (GSA-MD_v1), and following standard genotyping quality control, the genotyped data were imputed by use of chr1-22,X: Michigan Imputation Server v1.2.4 using Eagle v2.4 + minimac v4 and both 1000G phase3 (v5) in tgp.ph3/ and HRC v1.1 in hrc1.1/ as reference panels. Aortic stenosis case/control status was determined using the International Aortic Valve Genetics Consortium's definitions. A GWAS for aortic stenosis was performed in autosomes and the X chromosome using imputed data from 107 European genetic ancestry female cases and 4,407 European genetic ancestry female controls. The associations were modeled using logistic regression with adjustment for age and ancestry-specific principal components.

The Swedish Infrastructure for Medical Population-Based Life Course and Environmental Research (SIMPLER)

The Swedish Infrastructure for Medical Population-Based Life Course and Environmental Research (SIMPLER; https://www.simpler4health.se/) is an infrastructure that includes the SMC and COSM studies (see above) [10-13]. In addition to participants who have provided blood samples (see descriptions for SMC and COSM above), around 40,000 participants have provided a saliva sample to analyze human DNA and DNA from oral microbiota. Genotyping was performed using Illumina Infinium Global Screening Array version 3 (GSAv3), and following standard genotyping quality control and exclusion of participants overlapping with the clinical subcohorts, the genotyped data were imputed by use of chr1-22,X: Michigan Imputation Server v1.2.4 using Eagle v2.4 + minimac v4 and both 1000G phase3 (v5) in tgp.ph3/ and HRC v1.1 in hrc1.1/ as reference panels. Aortic stenosis case/control status was determined using the International Aortic Valve Genetics Consortium's provided definitions. A GWAS for aortic stenosis was performed in autosomes using imputed data from 1,023 European genetic ancestry cases and 24,387 European genetic ancestry controls. Additionally, in sex-stratified data, GWAS was performed for autosomes and the X-chromosome (853 male cases, 19,865 male controls; 170 female cases, 4,522 female controls). The associations were modeled using logistic regression with adjustment for age, sex (except sex-stratified analyses), and ancestry-specific principal components.

Supplemental Figures

Supplemental Figure 1: Schematic overview of study design. ***Legend*****:** a schematic overview of genome-wide association study analyses and secondary analyses. Abbreviations as follows, Chr: chromosome, MR: Mendelian randomization.

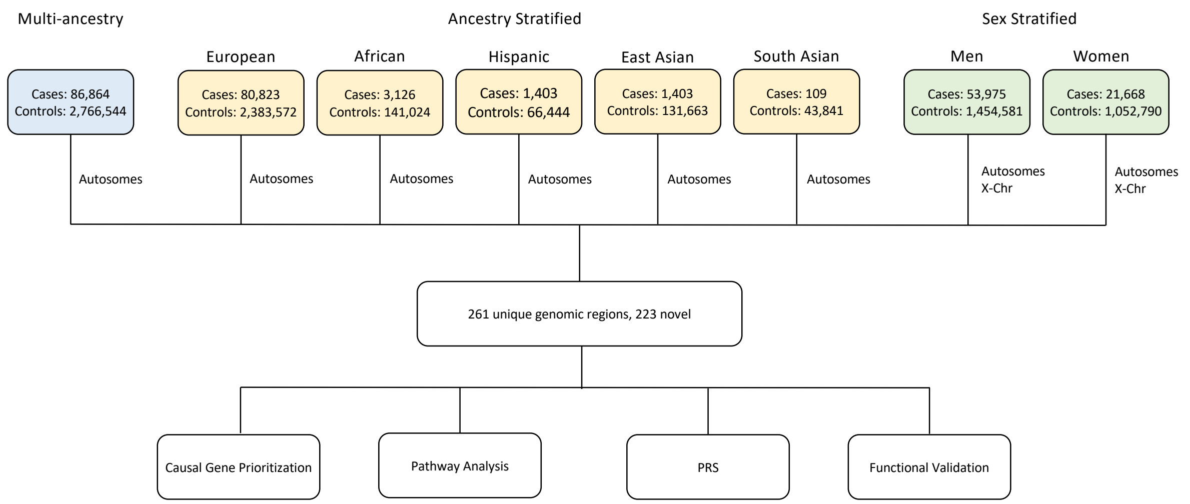

Supplemental Figure 2: Manhattan and QQ plot for multi-ancestry genome-wide association study of aortic stenosis in autosomes. ***Legend***: Manhattan plot (A) for our multi-ancestry genome wide association study of aortic stenosis in autosomes (N = 2,853,408 individuals). Significant variants are highlighted in green. Genome-wide significance (P-value = 5×10^{-8}) indicated by a horizontal red line. QQ plot (B) for our multi-ancestry genome wide association study of aortic stenosis in autosomes. Abbreviations as follows, $-\log_{10}(p)$: negative logarithm base 10 of p-value.

A: Manhattan plot

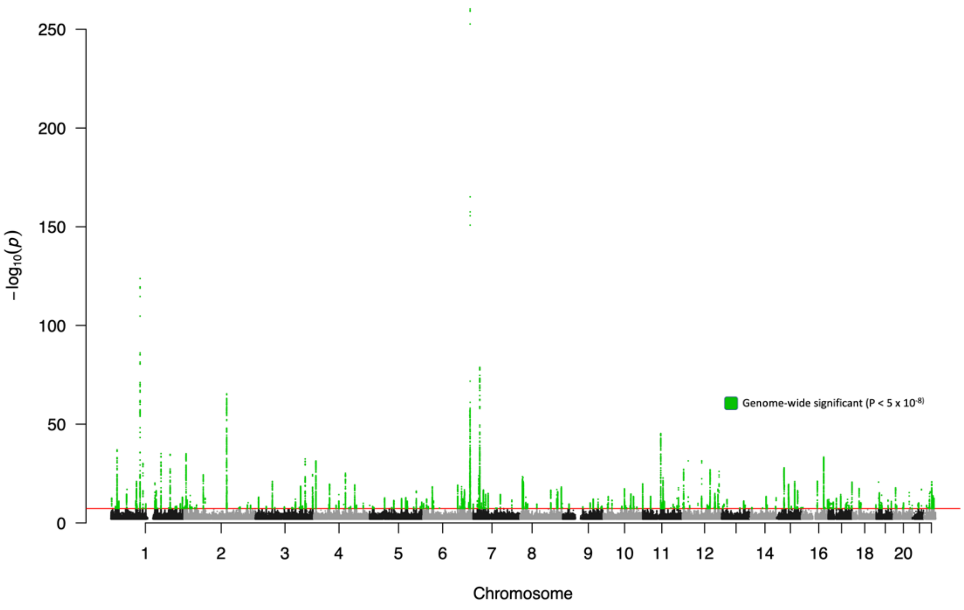

B: QQ plot

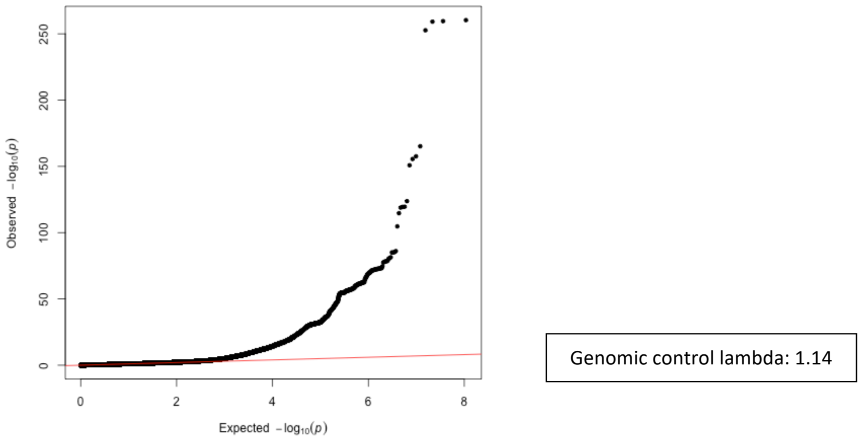

Supplemental Figure 3: Manhattan plot of multi-ancestry X-chromosome analysis. ***Legend*:** Manhattan plot for our multi-ancestry X-chromosome analysis of AS (N = 2,378,232 individuals). Significant variants highlighted in green. Genome-wide significant variants (P-value = 5×10^{-8}) indicated by a horizontal red line. Abbreviations as follows, $-\log_{10}(p)$: negative logarithm base 10 of p-value.

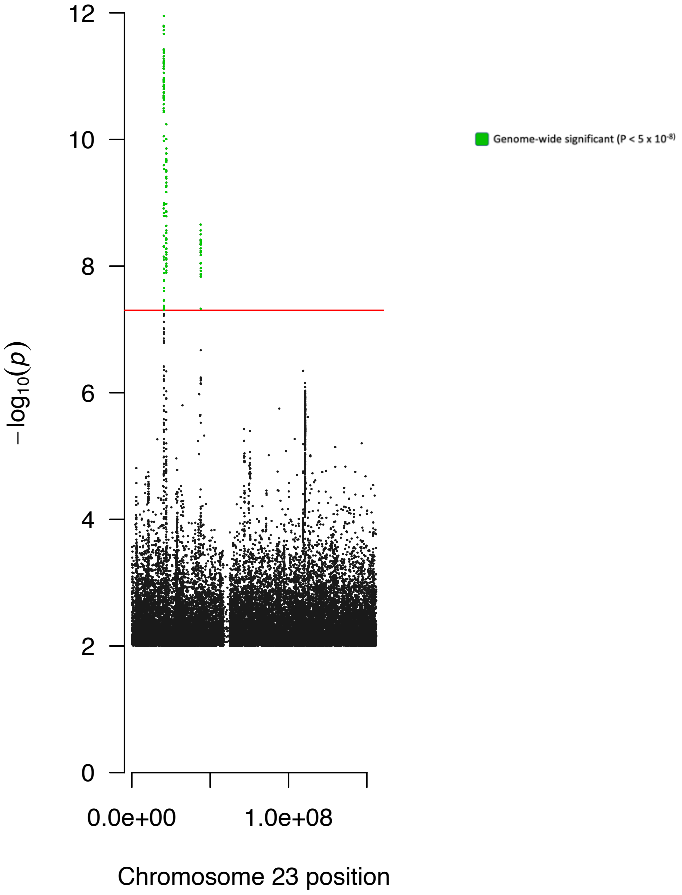

Supplemental Figure 4: Manhattan and QQ plot for European ancestry genome wide association study of aortic stenosis in autosomes (N = 2,464,395 individuals). ***Legend***: Manhattan plot (A) for our European ancestry genome wide association study of aortic stenosis in autosomes. Significant variants are highlighted in green. Genome-wide significance (P-value = 5×10^{-8}) indicated by a horizontal red line. QQ plot (B) for our European ancestry genome wide association study of aortic stenosis in autosomes. Abbreviations as follows, -$\log_{10}(p)$: negative logarithm base 10 of p-value.

A: Manhattan plot

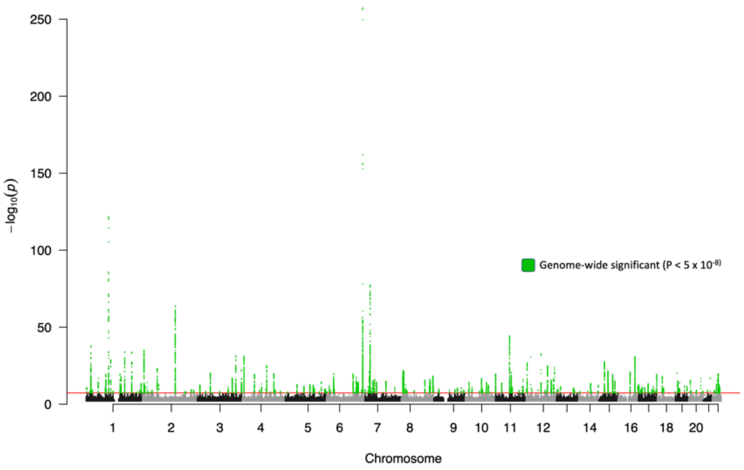

B: QQ plot

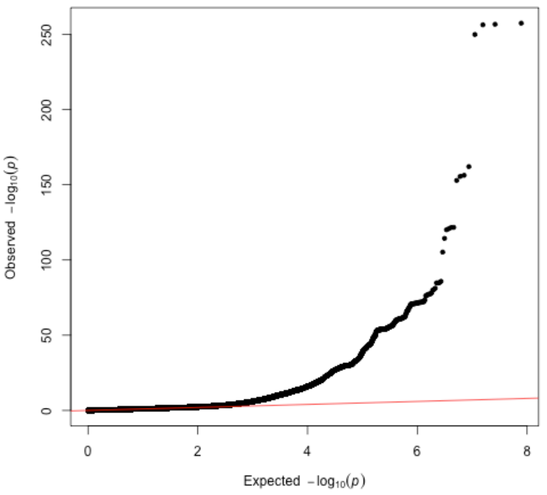

Genomic control lambda: 1.14

Supplemental Figure 5: Manhattan and QQ plot for African ancestry genome wide association study of aortic stenosis in autosomes (N = 144,150 individuals). ***Legend*:** Manhattan plot (A) for our African ancestry genome wide association study of aortic stenosis in autosomes. Significant loci are highlighted in green. Genome wide significance (P-value = 5×10^{-8}) indicated by a horizontal red line. QQ plot (B) for our African ancestry genome wide association study of aortic stenosis in autosomes. Abbreviations as follows, $-\log_{10}(p)$: negative logarithm base 10 of p-value.

A: Manhattan plot

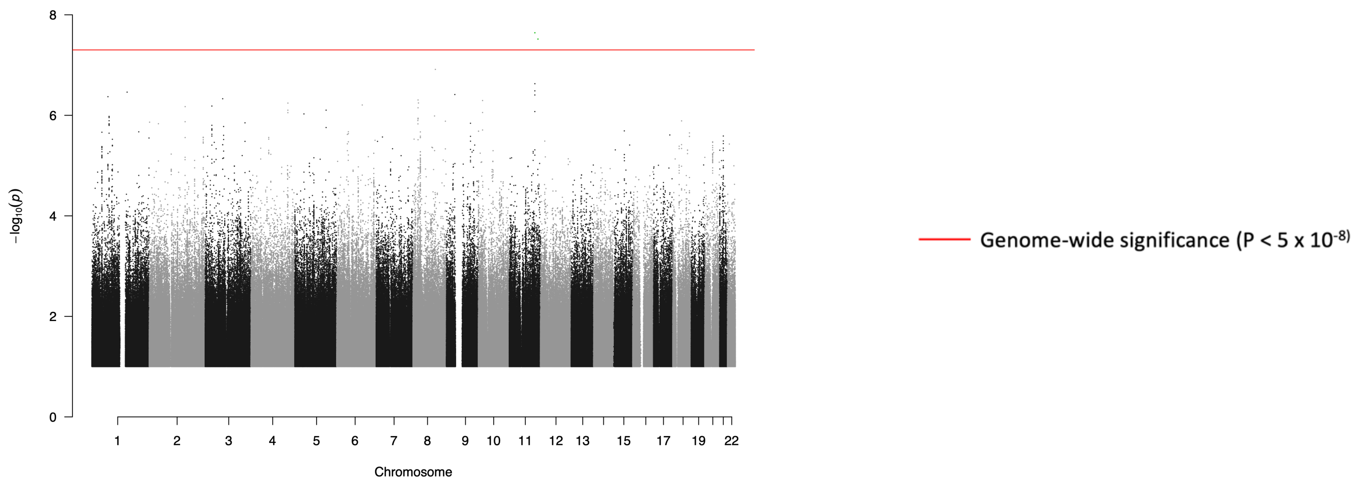

B: QQ plot

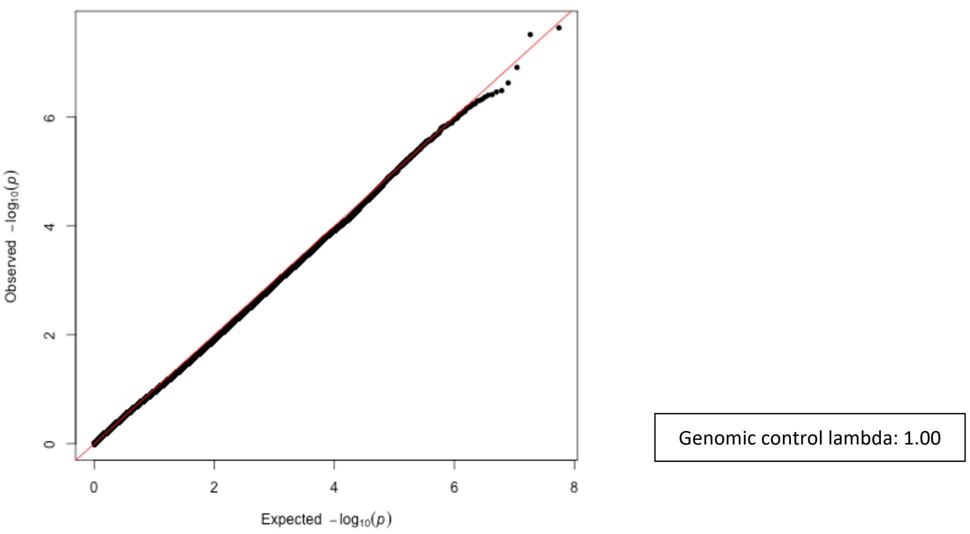

Supplemental Figure 6: Manhattan and QQ plot for Hispanic ancestry genome wide association study of aortic stenosis in autosomes (N = 66,444 individuals). ***Legend*:** Manhattan plot (A) for our Hispanic ancestry genome wide association study of aortic stenosis in autosomes. QQ plot (B) for our Hispanic ancestry genome wide association study of aortic stenosis in autosomes. Abbreviations as follows, $-\log_{10}(p)$: negative logarithm base 10 of P-value.

A: Manhattan plot

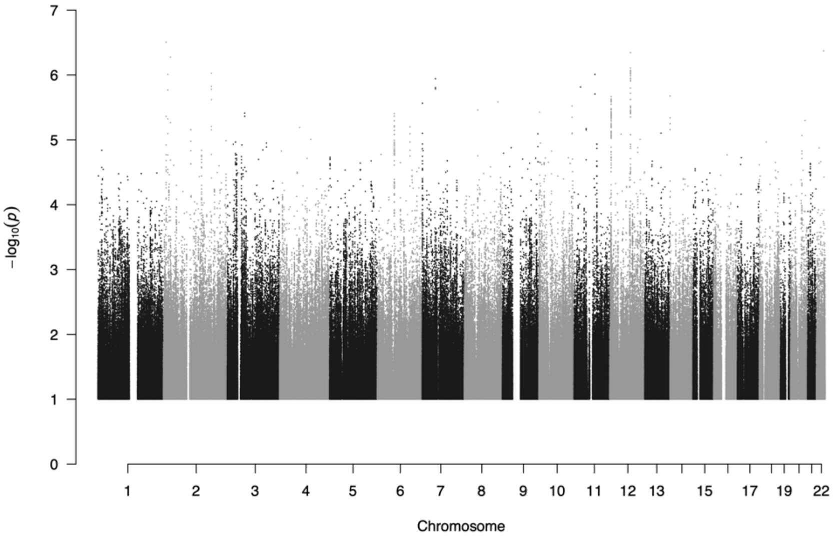

B: QQ plot

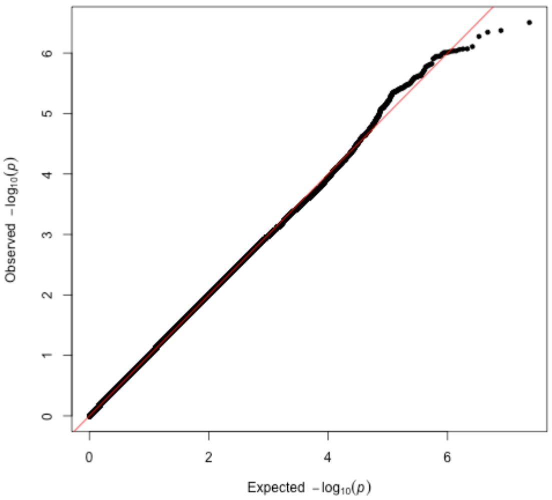

Genomic control lambda: 1.01

Supplemental Figure 7: Manhattan and QQ plot for East Asian ancestry genome wide association study of aortic stenosis in autosomes (N = 131,663 individuals). ***Legend*:** Manhattan plot (A) for our East Asian ancestry genome wide association study of aortic stenosis in autosomes. QQ plot (B) for our East Asian ancestry genome wide association study of aortic stenosis in autosomes. Abbreviations as follows, -$\log_{10}(p)$: negative logarithm base 10 of P-value.

A: Manhattan plot

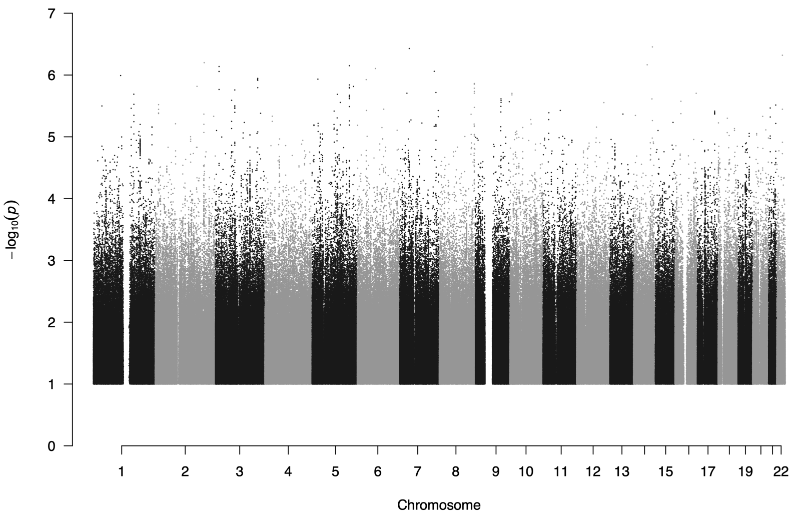

A: QQ plot

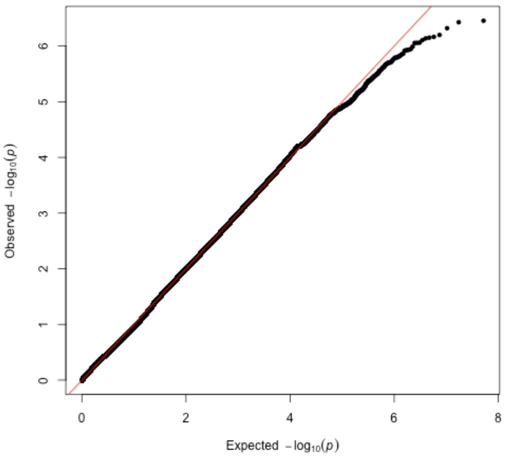

Genomic control lambda: 1.01

Supplemental Figure 8: Manhattan and QQ plot for South Asian ancestry genome wide association study of aortic stenosis in autosomes (N = 43,950 individuals). ***Legend*:** Manhattan plot (A) for our South Asian ancestry genome wide association study of aortic stenosis in autosomes. QQ plot (B) for our South Asian ancestry genome wide association study of aortic stenosis in autosomes. Abbreviations as follows, -$\log_{10}$(*p)*: negative logarithm base 10 of p-value.

A: Manhattan plot

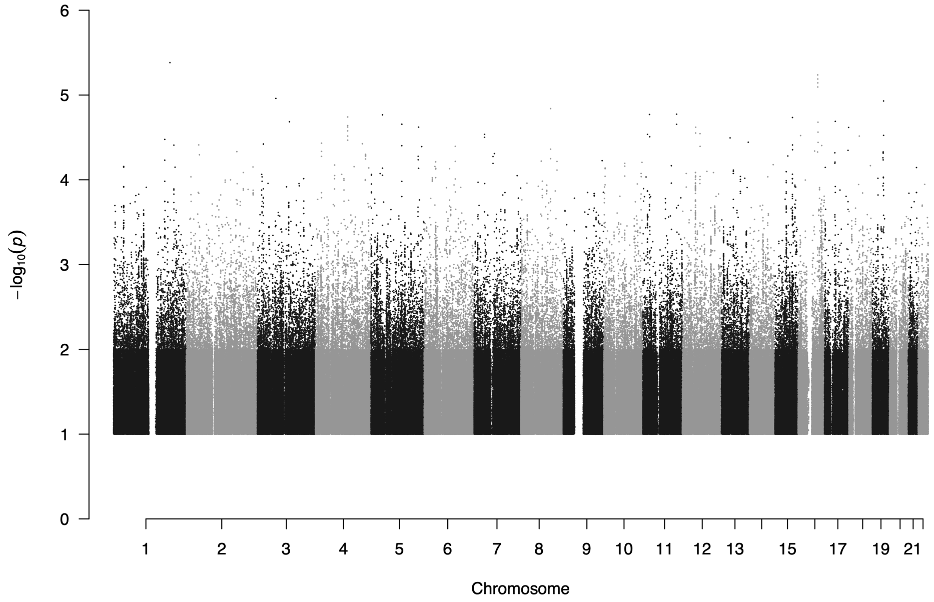

B: QQ plot

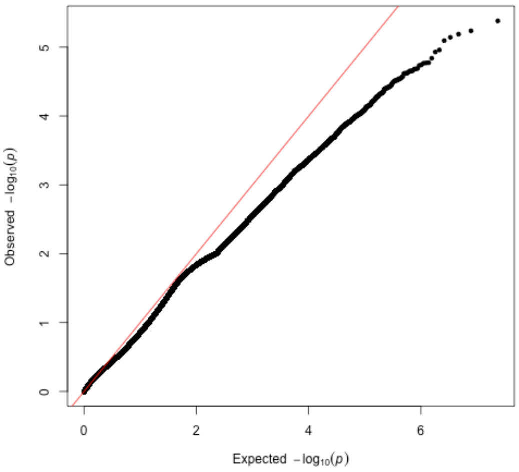

Genomic control lambda: 0.93

Supplemental Figure 9: Deming regression of multi-ancestry lead single nucleotide polymorphism effect estimates among African, Hispanic, East Asian, and South Asian ancestries. ***Legend***: Scatter plots of betas for multi-ancestry lead variants comparing results from specific ancestries (y-axis, AA: African ancestry, HA: Hispanic ancestry, EAS: East Asian ancestry, SAS: South Asian ancestry) and from multi-ancestry analysis (x-axis). Deming regression slope is shown with a blue line.

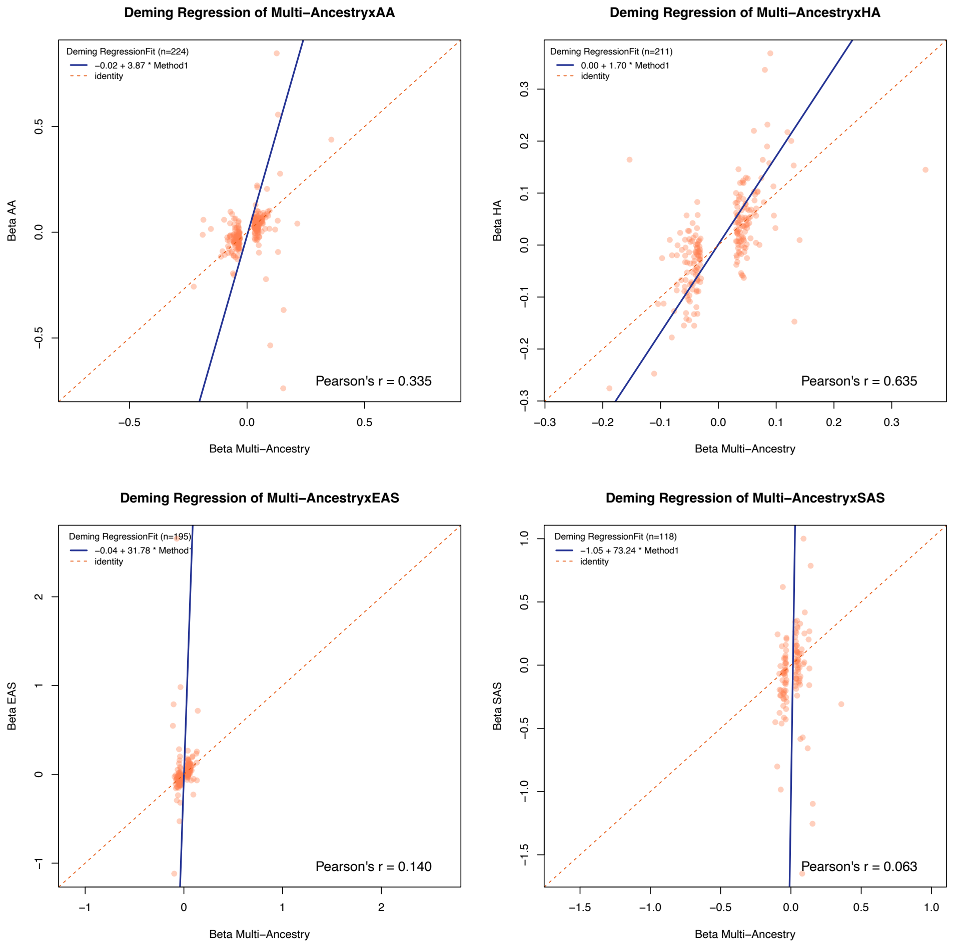

Supplemental Figure 10: Manhattan and QQ plot for female sex genome-wide association study of aortic stenosis in autosomes (N = 1,102,928 individuals). ***Legend***: Manhattan plot (A) for our female sex genome wide association study of aortic stenosis in autosomes. Significant loci are highlighted in green. Genome wide significance ($p = 5 \times 10^{-8}$) indicated by a horizontal red line. QQ plot (B) for our female sex genome wide association study of aortic stenosis in autosomes. Abbreviations as follows, $-\log_{10}(p)$: negative logarithm base 10 of p-value.

A: Manhattan plot

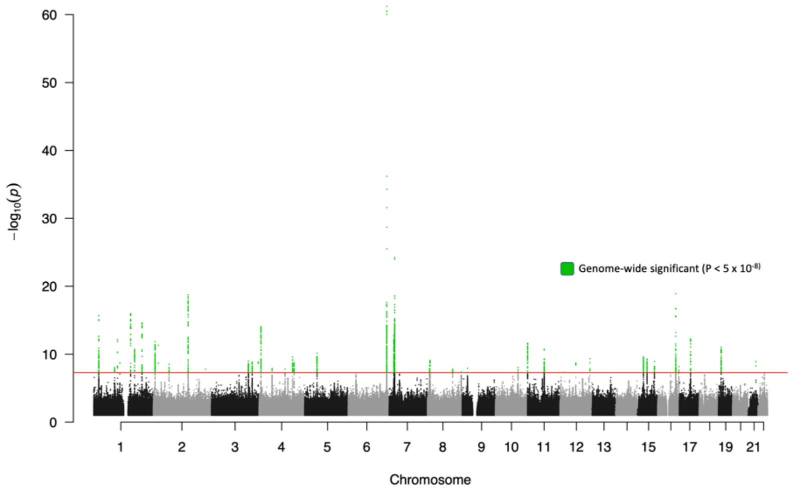

B: QQ plot

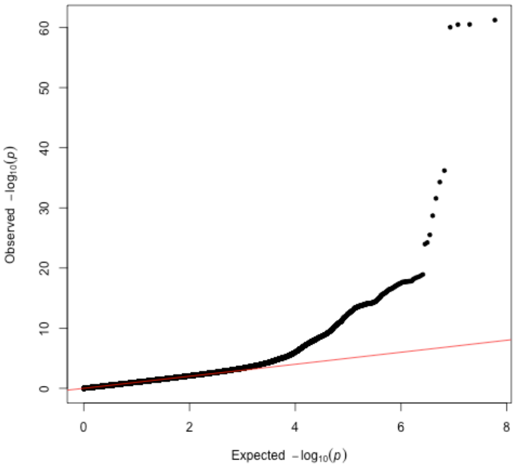

Genomic control lambda: 1.04

Supplemental Figure 11: Manhattan and QQ plot for male sex genome wide association study of aortic stenosis in autosomes (N = 1,526,601 individuals). **Legend:** Manhattan plot (A) for our male sex genome wide association study of aortic stenosis in autosomes. Significant loci are highlighted in green. Genome wide significance (p = $5x10^{-8}$) indicated by a horizontal red line. QQ plot (B) for our male sex GWAS of AS in autosomes. Abbreviations as follows, $-log_{10}(p)$: negative logarithm base 10 of p-value.

A: Manhattan plot

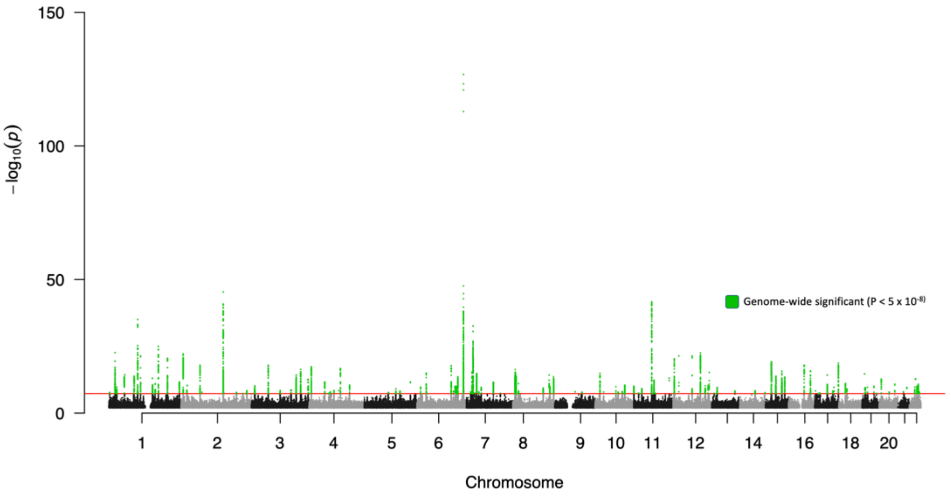

B: QQ plot

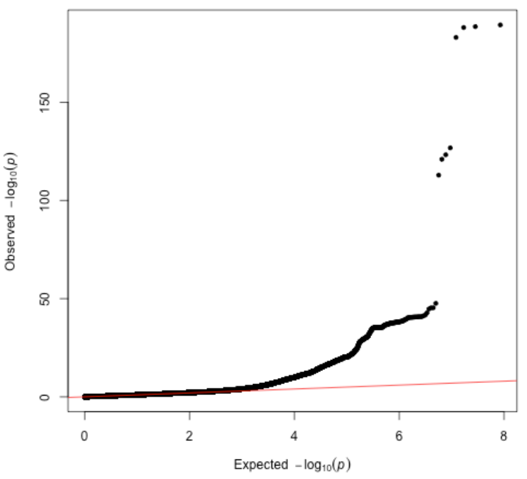

Genomic control lambda: 1.10

Supplemental Figure 12: Similarity matrix of representative ontologies for aortic stenosis.

Legend: Nodes indicate representative ontologies prioritized by DEPICT using multi-ancestry aortic stenosis genome wide association study summary data. Density of edges represents similarity between gene sets for each node (red).

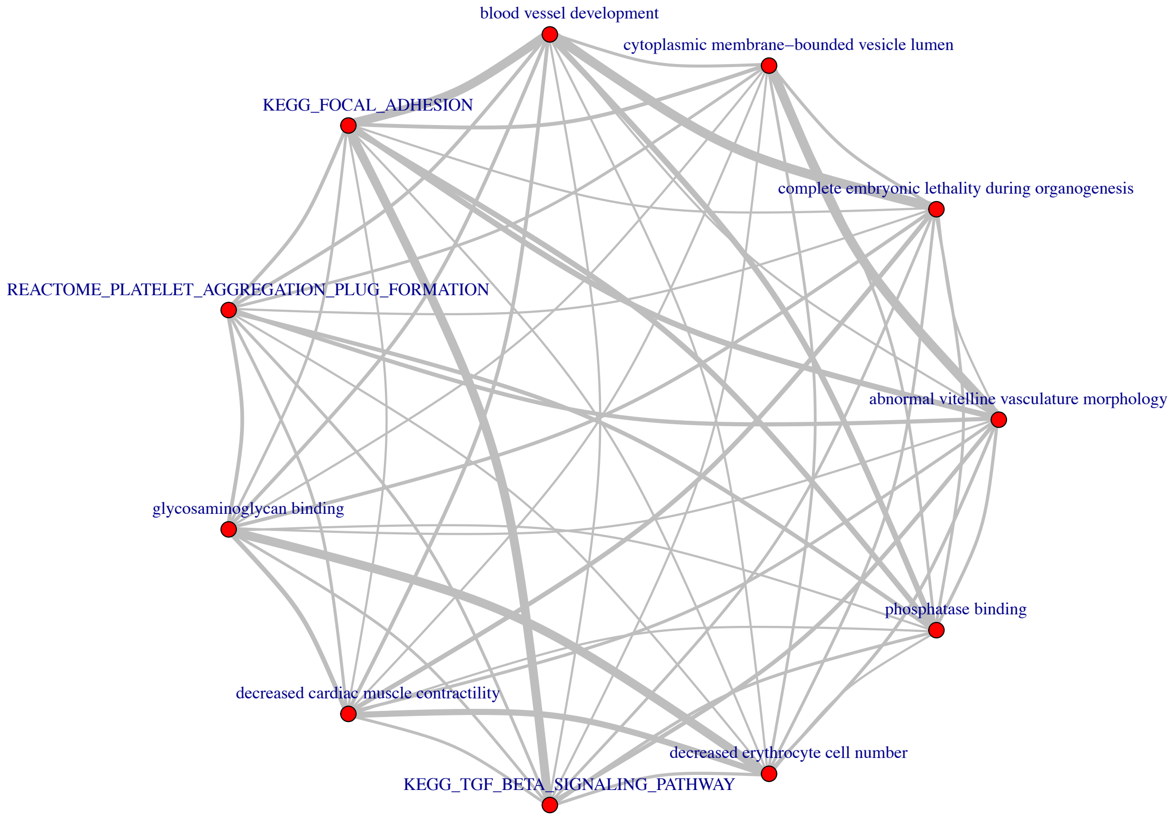

Supplemental Figure 13: Manhattan plot of phenome-wide association study results for aortic stenosis GWAS lead variants in the Million Veteran Program (N = 449,042 European genetic ancestry individuals). ***Legend*:** Manhattan plot with phenotype associations colored by trait type (see legend). Y-axis corresponds to the -log10(p-value) of the genotype-phenotype association. X-axis corresponds to the chromosomal position of the genotype. The top strongest associations are labeled by each lead variant's prioritized gene and the phenotype description.

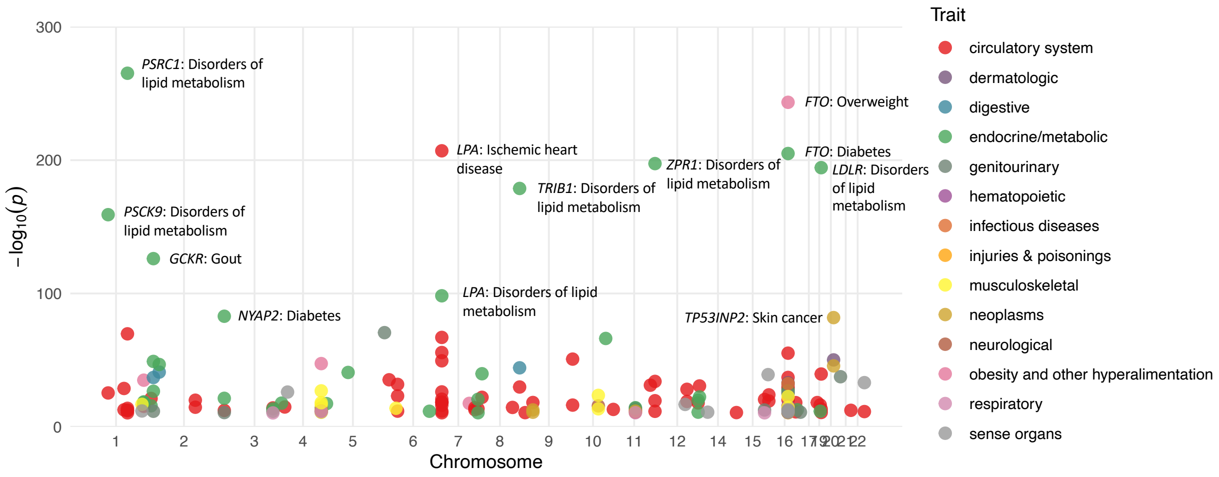

Supplemental Figure 14: Kaplan Meier curves depicting cumulative diagnosis of aortic stenosis by quintiles of genetic risk in the UK Biobank (N = 446,895) and TIMI trials (N = 59,866). ***Legend***: Kaplan Meier curves depicting cumulative diagnoses of aortic stenosis (Y-axis, in percent of each quintile) over time (X-axis) in the UKB (A) and TIMI clinical trials (B). Q1-Q5 represent quartiles 1 (lowest 20% genetic risk) to quartile 5 (highest 20% genetic risk).

A: UKB

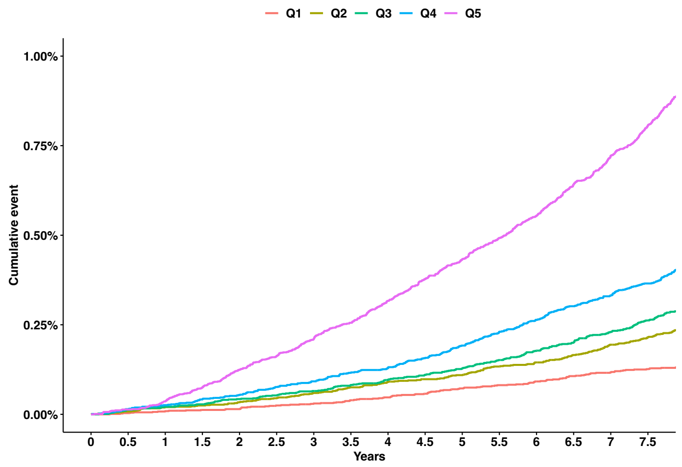

B: TIMI Clinical Trials

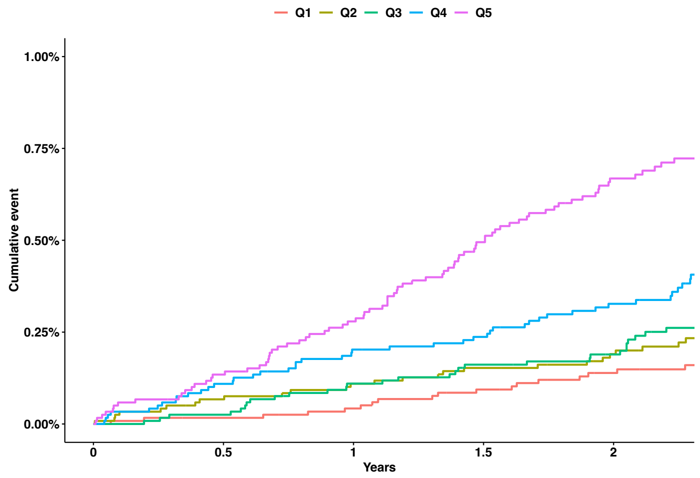

Supplemental Figure 15: Forest plot comparing the C-index for individual risk factors to a composite of clinical risk factors or clinical risk factors with genetic risk in the UK Biobank (N = 446,895) and TIMI trials (N = 59,866). ***Legend***: Forest plot indicating C-index for composite models and individual risk factors in risk estimation of aortic stenosis in the UK Biobank (A) and TIMI clinical trials (B). Abbreviations as follows: PRS, polygenic risk score; HTN, hypertension; BMI, body mass index; LDL-C, low density lipoprotein cholesterol; CAD, coronary artery disease; T2D, type 2 diabetes; CKD, chronic kidney disease.

A: UKB

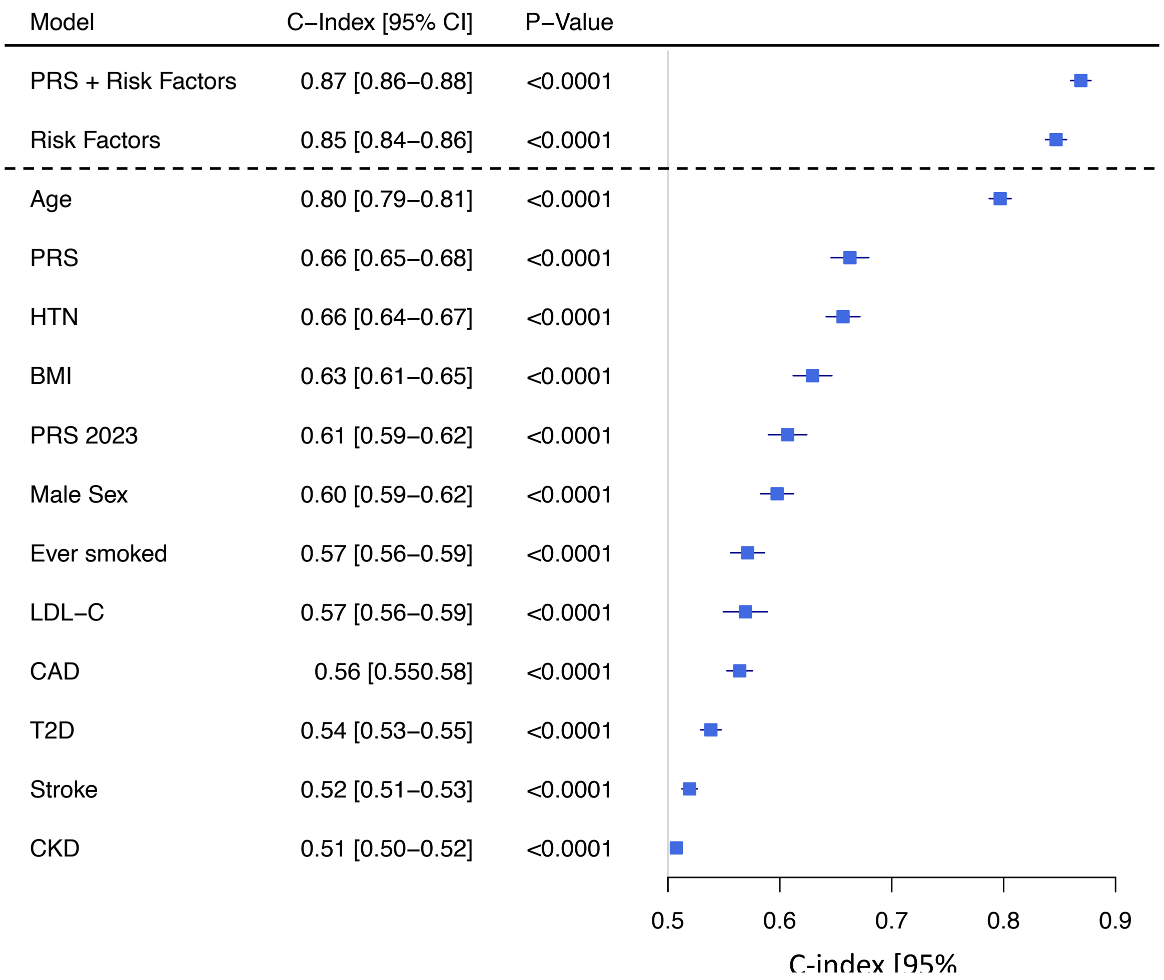

B: TIMI Clinical Trials

Model	C–Index [95% CI]	P–Value
PRS + Risk Factors	0.76 [0.72–0.79]	<0.0001
Risk Factors	0.71 [0.68–0.75]	<0.0001
Age	0.68 [0.65–0.72]	<0.0001
PRS	0.66 [0.62–0.69]	<0.0001
PRS 2023	0.60 [0.56–0.63]	<0.0001
T2D	0.56 [0.53–0.59]	<0.0001
LDL–C	0.55 [0.50–0.60]	<0.0001
BMI	0.55 [0.51–0.59]	<0.0001
HLD	0.54 [0.51–0.57]	<0.0001
Ever smoked	0.53 [0.51–0.55]	<0.0001
HTN	0.53 [0.51–0.55]	<0.0001
Male sex	0.52 [0.49–0.55]	<0.0001
eGFR	0.50 [0.49–0.51]	<0.0001

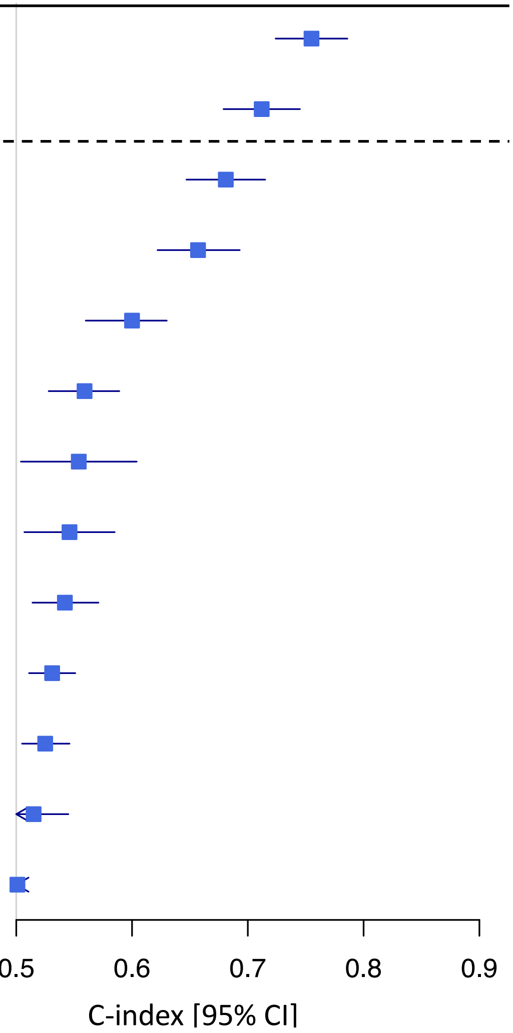

Author Funding and Acknowledgements:

R.K. is supported by the Japan Agency for Medical Research, Development (JP24ek0210164, JP23km0405209, JP23km0405209, JP23bm1423005). I.K. was supported by a Grant-in-Aid for Scientific Research (S) of the Japan Society for the Promotion of Science and by the Japan Agency for Medical Research, Development (JP20ek0210152, JP18gm6210010, JP20ek0210141, JP20ek0109440, JP20ek0109487, JP17gm0810013, JP18km0405209, JP21ek0109543, JP21ek0109569, JP21tm0724601, JP22ama121016, JP22ek0210172, JP22ek0210167, JP22bm1123011). K.M.B. is funded by NIH T32 grant 5T32HL007895. J.J.S. is funded by ANR [13-BSV6-0011], FRM [DCV20070409278] and International research programs (IRP) VERACITIES, an I-SITE NExT health and engineering initiative (Ecole Centrale & Nantes University) and IRP GAINES, an Inserm & CNRS grant. We thank Marie Marrec and Guénola Coste for their contribution to clinical data collection for Nantes/CAVS France and we are most grateful to the Genomics Core Facility GenoA, member of Biogenouest and France Genomique and to the Bioinformatics Core Facility BiRD, member of Biogenouest and Institut Français de Bioinformatique (IFB) (ANR-11-INBS-0013) for the use of their resources and their technical support. We thank the biological resource centre for biobanking (CHU Nantes, Nantes Université, Centre de ressources biologiques (BB-0033-00040), F-44000 Nantes, France). A.G. is supported by the Academy of Finland (grant no. 323116) and by the European Research Council under the European Union's Horizon 2020 Research and Innovation Programme (grant no. 945733). S.F., D.A.V.H.: Genes & Health is/has recently been core-funded by Wellcome (WT102627, WT210561), the Medical Research Council (UK) (M009017, MR/X009777/1, MR/X009920/1), Higher Education Funding Council for England Catalyst, Barts Charity (845/1796), Health Data

Research UK (for London substantive site), and research delivery support from the NHS National Institute for Health Research Clinical Research Network (North Thames). We acknowledge the support of the National Institute for Health and Care Research Barts Biomedical Research Centre (NIHR203330); a delivery partnership of Barts Health NHS Trust, Queen Mary University of London, St George's University Hospitals NHS Foundation Trust and St George's University of London. Genes & Health is/has recently been funded by Alnylam Pharmaceuticals, Genomics PLC; and a Life Sciences Industry Consortium of AstraZeneca PLC, Bristol-Myers Squibb Company, GlaxoSmithKline Research and Development Limited, Maze Therapeutics Inc, Merck Sharp & Dohme LLC, Novo Nordisk A/S, Pfizer Inc, Takeda Development Centre Americas Inc. We thank Social Action for Health, Centre of The Cell, members of our Community Advisory Group, and staff who have recruited and collected data from volunteers. We thank the NIHR National Biosample Centre (UK Biocentre), the Social Genetic & Developmental Psychiatry Centre (King's College London), Wellcome Sanger Institute, and Broad Institute for sample processing, genotyping, sequencing and variant annotation. This work uses data provided by patients and collected by the NHS as part of their care and support. This research utilised Queen Mary University of London's Apocrita HPC facility, supported by QMUL Research-IT, http://doi.org/10.5281/zenodo.438045. We thank: Barts Health NHS Trust, NHS Clinical Commissioning Groups (City and Hackney, Waltham Forest, Tower Hamlets, Newham, Redbridge, Havering, Barking and Dagenham), East London NHS Foundation Trust, Bradford Teaching Hospitals NHS Foundation Trust, Public Health England (especially David Wyllie), Discovery Data Service/Endeavour Health Charitable Trust (especially David Stables), Voror Health Technologies Ltd (especially Sophie Don), NHS England (for what was NHS Digital) - for GDPR-compliant data

sharing backed by individual written informed consent. Most of all we thank all of the volunteers participating in Genes & Health. A favourable ethical opinion for the main Genes & Health research study was granted by NRES Committee London - South East (reference 14/LO/1240) on 16 Sept 2014. Queen Mary University of London is the Sponsor, and Data Controller. J.S. is supported by Deutsche Forschungsgemeinschaft (DFG) (TRR259). T.T. received funding form the Deutsche Herzstiftung (FKZ F/21/20). B.M.B. is funded by The Liaison Committee for education, research and innovation in Central Norway; and the Joint Research Committee between St. Olavs Hospital and the Faculty of Medicine and Health Sciences, NTNU. The genotyping in HUNT was financed by the National Institute of Health (NIH); University of Michigan; The Research Council of Norway; The Liaison Committee for education, research and innovation in Central Norway; and the Joint Research Committee between St. Olavs Hospital and the Faculty of Medicine and Health Sciences, NTNU. M.S.Se. is supported by TOPMed (2022-6842.02). S.K. is supported by NHLBI (K99HL169733). M.G.L. is supported by the Doris Duke Foundation (Award 2023-0224). K.B.: The PROCAM-2 Study was initiated and conducted by the Leibniz Institute for Arteriosclerosis Research (LIFA) at the University of Münster, under the leadership of Prof. Dr. G. Assmann. After his retirement all data were transferred to the University of Münster for further scientific use. DNA isolation was done later with funds from the dean of the medical faculty. Genotyping was enabled through funds from the German Center for Cardiovascular Disease (DZHK) and the written follow-up with funds from the Institute of Epidemiology and Social Medicine University of Münster. C.T.R. reports research grants through Brigham and Women's Hospital from Anthos, AstraZeneca, Daiichi Sankyo, Janssen, and Novartis and has received honoraria for scientific advisory boards and consulting from Anthos, Bayer, Bristol Myers Squibb,

Daiichi Sankyo, Janssen, and Pfizer. He is also a member of the TIMI Study Group which as received institutional research grant support through Brigham and Women's Abbott, Abiomed, Inc., Amgen, Anthos Therapeutics, ARCA Biopharma, Inc., AstraZeneca, Boehringer Ingelheim, Daiichi-Sankyo, Ionis Pharmaceuticals, Inc., Janssen Research and Development, LLC, MedImmune, Merck, Novartis, Pfizer, Regeneron Pharmaceuticals, Inc., Roche, Saghmos Therapeutics, Inc., Siemens Healthcare Diagnostics, Inc., Softcell Medical Limited, The Medicines Company, Verve Therapeutics, Inc., and Zora Biosciences. K.I. was supported by a Grant-in-Aid for Scientific Research (B) of the Japan Society for the Promotion of Science, the Japan Agency for Medical Research, Development (JP24ek0210164, JP23km0405209, JP23km0405209, JP23bm1423005), Research Funding for Longevity Sciences from the NCGG, and AMED JP25gm7010001. R.D. is supported by the National Institute of General Medical Sciences of the NIH (R35-GM124836). Recruitment into the GeneCast study was supported by the NIHR Leicester Biomedical Research Centre (BRC-1215-20010), who also supported R.D., M.D.M. and P.S.B. C.P.N. and N.J.S. were funded by the British Heart Foundation (CH/03/001). T.L.T. is supported by Fédération Française de Cardiologie and Fondation Coeur et Recherche grants. D.M.Z. is supported by PHRC National 2005 and 2010, and PHRCI 2007. C.G.: CCPM is supported by UCHealth and the University of Colorado School of Medicine. S.C.L. was supported by grants from the Swedish Heart-Lung Foundation (Hjärt-Lungfonden; 20190247), the Swedish Research Council (Vetenskapsrådet; 2019-00977), and the Swedish Cancer Society (Cancerfonden). Ka.M.: SMC and COSM are cohorts of the national research infrastructure SIMPLER, which receives funding through the Swedish Research Council under grant No. 2017-00644, 2019-01291 and 2021-00160 (to Uppsala University and Karl Michaëlsson). P.M. is supported by Canadian

Institute of Health Reseach (FRN159697, FRN191807) and is the recipient of the Joseph C. Edwards Foundation granted to Université Laval. J.G.S. was supported by grants from the Swedish Heart-Lung Foundation (2022-0344, 2022-0345), the Swedish Research Council (2021-02273), the European Research Council (ERC-STG-2015-679242), Gothenburg University, Skåne University Hospital, governmental funding of clinical research within the Swedish National Health Service, a generous donation from the Knut and Alice Wallenberg foundation to the Wallenberg Center for Molecular Medicine in Lund, and funding from the Swedish Research Council (Linnaeus grant Dnr 349-2006-237, Strategic Research Area Exodiab Dnr 2009-1039) and Swedish Foundation for Strategic Research (Dnr IRC15-0067) to the Lund University Diabetes Center. S.S.: Swedish Heart and Lung Fundation, Umeå University, County council in Västerbotten. N.A.M. receives grant support from the National Institutes of Health and involvement in clinical trials with Amgen, Ionis, Pfizer, Novartis, and AstraZeneca. Speaking honoraria from Amgen. M.S.Sa. reports research grant support through Brigham and Women's Hospital from: Abbott; Amgen; Anthos Therapeutics, Inc.; AstraZeneca; Boehringer Ingelheim; Daiichi-Sankyo; Ionis; Merck; Novartis; Pfizer; Saghmos Therapeutics; Verve Therapeutics, consulting for: Amgen; Anthos Therapeutics, Inc.; AstraZeneca; Beren Therapeutics; Boehringer Ingelheim; Dr. Reddy's Laboratories; Fibrogen; Merck; Moderna; Novo Nordisk; Precision BioSciences; Silence Therapeutics, and honoraria from Boehringer Ingelheim and from AstraZeneca. B.P.: We gratefully acknowledge the support of the Institute for Precision Health, participating patients from the UCLA ATLAS Precision Health Biobank, UCLA David Geffen School of Medicine, UCLA Clinical and Translational Science Institute grant number UL1TR001881, and UCLA Health. E.A., T.E. received funding from the European Union through Horizon 2020 research and innovation

program under grants no. 810645, 894987, 101137201 and 101137154, through the European Regional Development Fund project MOBEC008 and Estonian Research Council Grant PRG1291. We want to acknowledge the participants of the Estonian Biobank for their contributions. The Estonian Biobank Research Team participated in data collection, genotyping, QC and imputation, and consists of Prof. Andres Metspalu, Prof. Lili Milani, Prof. Reedik Mägi, Mari Nelis and Georgi Hudjashov. The analyses were partially carried out in the High Performance Computing Center, University of Tartu. The activities of the EstBB are regulated by the Human Genes Research Act, which was adopted in 2000 specifically for the operations of EstBB. Individual level data analysis in EstBB was carried out under ethical approval 1.1-12/624 from the Estonian Committee on Bioethics and Human Research (Estonian Ministry of Social Affairs), using data according to release application 6-7/GI/16274 from the Estonian Biobank. The Trøndelag Health Study (The HUNT Study) is a collaboration between HUNT Research Centre (Faculty of Medicine and Health Sciences, NTNU, Norwegian University of Science and Technology), Trøndelag County Council, Central Norway Regional Health Authority, and the Norwegian Institute of Public Health. The genotyping in HUNT was financed by the National Institutes of Health; University of Michigan; the Research Council of Norway; the Liaison Committee for Education, Research and Innovation in Central Norway; and the Joint Research Committee between St Olavs hospital and the Faculty of Medicine and Health Sciences, NTNU. The genetic investigations of the HUNT Study are a collaboration between researchers from the HUNT Center for Molecular and Clinical Epidemiology (formerly known as the K.G. Jebsen Center for Genetic Epidemiology as of August 1st 2023), NTNU, and the University of Michigan Medical School and the University of Michigan School of Public Health. We thank HUNT participants for donating their time, samples, and

information to help others; clinicians and other employees at Nord-Trøndelag Hospital Trust for their support and for contributing to data collection. This research is based on data from the Million Veteran Program, Office of Research and Development, Veterans Health Administration, and was supported by award #BX004821. This publication does not represent the views of the Department of Veteran Affairs or the United States Government.

VA Million Veteran Program – Core Acknowledgements for Publications

MVP Program Office

- Sumitra Muralidhar, Ph.D., Program Director
  US Department of Veterans Affairs, 810 Vermont Avenue NW, Washington, DC 20420
- Jennifer Moser, Ph.D., Associate Director, Scientific Programs
  US Department of Veterans Affairs, 810 Vermont Avenue NW, Washington, DC 20420
- Jennifer E. Deen, B.S., Associate Director, Cohort & Public Relations
  US Department of Veterans Affairs, 810 Vermont Avenue NW, Washington, DC 20420

MVP Executive Committee

- Co-Chair: Philip S. Tsao, Ph.D.
  VA Palo Alto Health Care System, 3801 Miranda Avenue, Palo Alto, CA 94304
- Co-Chair: Sumitra Muralidhar, Ph.D.
  US Department of Veterans Affairs, 810 Vermont Avenue NW, Washington, DC 20420
- J. Michael Gaziano, M.D., M.P.H.
  VA Boston Healthcare System, 150 S. Huntington Avenue, Boston, MA 02130
- Elizabeth Hauser, Ph.D.
  Durham VA Medical Center, 508 Fulton Street, Durham, NC 27705

- Amy Kilbourne, Ph.D., M.P.H.

VA HSR&D, 2215 Fuller Road, Ann Arbor, MI 48105

- Michael Matheny, M.D., M.S., M.P.H.

VA Tennessee Valley Healthcare System, 1310 24th Ave. South, Nashville, TN 37212

- Dave Oslin, M.D.

Philadelphia VA Medical Center, 3900 Woodland Avenue, Philadelphia, PA 19104

- Deepak Voora, MD

Durham VA Medical Center, 508 Fulton Street, Durham, NC 27705

<u>MVP Co-Principal Investigators</u>

- J. Michael Gaziano, M.D., M.P.H.

VA Boston Healthcare System, 150 S. Huntington Avenue, Boston, MA 02130

- Philip S. Tsao, Ph.D.

VA Palo Alto Health Care System, 3801 Miranda Avenue, Palo Alto, CA 94304

<u>MVP Core Operations</u>

- Jessica V. Brewer, M.P.H., Director, MVP Cohort Operations

VA Boston Healthcare System, 150 S. Huntington Avenue, Boston, MA 02130

- Mary T. Brophy M.D., M.P.H., Director, VA Central Biorepository

VA Boston Healthcare System, 150 S. Huntington Avenue, Boston, MA 02130

- Kelly Cho, M.P.H, Ph.D., Director, MVP Phenomics

VA Boston Healthcare System, 150 S. Huntington Avenue, Boston, MA 02130

- Lori Churby, B.S., Director, MVP Regulatory Affairs

VA Palo Alto Health Care System, 3801 Miranda Avenue, Palo Alto, CA 94304

- Scott L. DuVall, Ph.D., Director, VA Informatics and Computing Infrastructure (VINCI)

  VA Salt Lake City Health Care System, 500 Foothill Drive, Salt Lake City, UT 84148

- Saiju Pyarajan Ph.D., Director, Data and Computational Sciences

  VA Boston Healthcare System, 150 S. Huntington Avenue, Boston, MA 02130

- Robert Ringer, Pharm.D., Director, VA Albuquerque Central Biorepository

  New Mexico VA Health Care System, 1501 San Pedro Drive SE, Albuquerque, NM 87108

- Luis E. Selva, Ph.D., Director, MVP Biorepository Coordination

  VA Boston Healthcare System, 150 S. Huntington Avenue, Boston, MA 02130

- Shahpoor (Alex) Shayan, M.S., Director, MVP PRE Informatics

  VA Boston Healthcare System, 150 S. Huntington Avenue, Boston, MA 02130

- Brady Stephens, M.S., Principal Investigator, MVP Information Center Canandaigua

  VA Medical Center, 400 Fort Hill Avenue, Canandaigua, NY 14424

- Stacey B. Whitbourne, Ph.D., Director, MVP Cohort Development and Management

  VA Boston Healthcare System, 150 S. Huntington Avenue, Boston, MA 02130

References

1. All of Us Research Program, et al. The "All of Us" Research Program. *N Engl J Med*. 2019;381:668-676. doi: 10.1056/NEJMsr1809937

2. Nagai A, et al. Overview of the BioBank Japan Project: Study design and profile. *J Epidemiol*. 2017;27:S2-S8. doi: 10.1016/j.je.2016.12.005

3. Belbin GM, et al. Toward a fine-scale population health monitoring system. *Cell*. 2021;184:2068-2083 e2011. doi: 10.1016/j.cell.2021.03.034

4. Roden DM, et al. Development of a large-scale de-identified DNA biobank to enable personalized medicine. *Clin Pharmacol Ther*. 2008;84:362-369. doi: 10.1038/clpt.2008.89

5. Kraus WE, et al. A Guide for a Cardiovascular Genomics Biorepository: the CATHGEN Experience. *J Cardiovasc Transl Res*. 2015;8:449-457. doi: 10.1007/s12265-015-9648-y

6. Theriault S, et al. Genetic Association Analyses Highlight IL6, ALPL, and NAV1 As 3 New Susceptibility Genes Underlying Calcific Aortic Valve Stenosis. *Circ Genom Precis Med*. 2019;12:e002617. doi: 10.1161/CIRCGEN.119.002617

7. Cimadevilla C, et al. Prognostic value of B-type natriuretic peptide in elderly patients with aortic valve stenosis: the COFRASA-GENERAC study. *Heart*. 2013;99:461-467. doi: 10.1136/heartjnl-2012-303284

8. Dougados M, et al. The DESIR cohort: a 10-year follow-up of early inflammatory back pain in France: study design and baseline characteristics of the 708 recruited patients. *Joint Bone Spine*. 2011;78:598-603. doi: 10.1016/j.jbspin.2011.01.013

9. Wiley LK, et al. Building a vertically integrated genomic learning health system: The biobank at the Colorado Center for Personalized Medicine. *Am J Hum Genet*. 2024;111:11-23. doi: 10.1016/j.ajhg.2023.12.001

10. Warensjo Lemming E, et al. Meat consumption and the risk of hip fracture in women and men: two prospective Swedish cohort studies. *Eur J Nutr*. 2024. doi: 10.1007/s00394-024-03385-z

11. Michaelsson K, et al. Milk intake and risk of mortality and fractures in women and men: cohort studies. *BMJ*. 2014;349:g6015. doi: 10.1136/bmj.g6015

12. Michaelsson K, et al. Combined associations of body mass index and adherence to a Mediterranean-like diet with all-cause and cardiovascular mortality: A cohort study. *PLoS Med*. 2020;17:e1003331. doi: 10.1371/journal.pmed.1003331

13. Yuan S, et al. Anti-inflammatory diet and incident peripheral artery disease: Two prospective cohort studies. *Clin Nutr*. 2022;41:1191-1196. doi: 10.1016/j.clnu.2022.04.002

14. Sorensen E, et al. Data Resource Profile: The Copenhagen Hospital Biobank (CHB). *Int J Epidemiol*. 2021;50:719-720e. doi: 10.1093/ije/dyaa157

15. Laursen IH, et al. Cohort profile: Copenhagen Hospital Biobank - Cardiovascular Disease Cohort (CHB-CVDC): Construction of a large-scale genetic cohort to facilitate a better understanding of heart diseases. *BMJ Open*. 2021;11:e049709. doi: 10.1136/bmjopen-2021-049709

16. Erikstrup C, et al. Cohort Profile: The Danish Blood Donor Study. *Int J Epidemiol*. 2023;52:e162-e171. doi: 10.1093/ije/dyac194

17. Gudbjartsson DF, et al. Large-scale whole-genome sequencing of the Icelandic population. *Nat Genet*. 2015;47:435-444. doi: 10.1038/ng.3247

18. Bulik-Sullivan BK, et al. LD Score regression distinguishes confounding from polygenicity in genome-wide association studies. *Nat Genet*. 2015;47:291-295. doi: 10.1038/ng.3211

19. Leitsalu L, et al. Cohort Profile: Estonian Biobank of the Estonian Genome Center, University of Tartu. *Int J Epidemiol*. 2015;44:1137-1147. doi: 10.1093/ije/dyt268

20. Kurki MI, et al. Finngen provides genetic insights from a well-phenotyped isolated population. *Nature*. 2023;613:508-518. doi: 10.1038/s41586-022-05473-8

21. Kvale MN, et al. Genotyping Informatics and Quality Control for 100,000 Subjects in the Genetic Epidemiology Research on Adult Health and Aging (GERA) Cohort. *Genetics*. 2015;200:1051-1060. doi: 10.1534/genetics.115.178905

22. Finer S, et al. Cohort Profile: East London Genes & Health (ELGH), a community-based population genomics and health study in British Bangladeshi and British Pakistani people. *Int J Epidemiol*. 2020;49:20-21i. doi: 10.1093/ije/dyz174

23. Brumpton BM, et al. The HUNT study: A population-based cohort for genetic research. *Cell Genom*. 2022;2:100193. doi: 10.1016/j.xgen.2022.100193

24. Taylor DP. HerediGene Population Study IT infrastructure: A model to support genomic research recruitment and precision public health. *AMIA Annu Symp Proc*. 2023:689-698.

25. Eggertsson HP, et al. Graphtyper enables population-scale genotyping using pangenome graphs. *Nat Genet*. 2017;49:1654-1660. doi: 10.1038/ng.3964

26. Berglund G, Elmstahl S, Janzon L, Larsson SA. The Malmo Diet and Cancer Study. Design and feasibility. *J Intern Med*. 1993;233:45-51. doi: 10.1111/j.1365-2796.1993.tb00647.x

27. Koyama S, et al. Decoding Genetics, Ancestry, and Geospatial Context for Precision Health. *medRxiv*. 2023.

28. Gaziano JM, et al. Million Veteran Program: A mega-biobank to study genetic influences on health and disease. *J Clin Epidemiol*. 2016;70:214-223. doi: 10.1016/j.jclinepi.2015.09.016

29. Verma A, et al. The Penn Medicine BioBank: Towards a Genomics-Enabled Learning Healthcare System to Accelerate Precision Medicine in a Diverse Population. *J Pers Med*. 2022;12. doi: 10.3390/jpm12121974

30. Sudlow C, et al. UK biobank: an open access resource for identifying the causes of a wide range of complex diseases of middle and old age. *PLoS Med*. 2015;12:e1001779. doi: 10.1371/journal.pmed.1001779

31. The 1000 Genomes Project Consortium, et al. A global reference for human genetic variation. *Nature*. 2015;526:68-74. doi: 10.1038/nature15393

32. Huang J, et al. Improved imputation of low-frequency and rare variants using the UK10K haplotype reference panel. *Nat Commun*. 2015;6:8111. doi: 10.1038/ncomms9111

33. Ljungberg J, et al. Proteomic Biomarkers for Incident Aortic Stenosis Requiring Valvular Replacement. *Circulation*. 2018;138:590-599. doi: 10.1161/CIRCULATIONAHA.117.030414

34. Ljungberg J, et al. Traditional Cardiovascular Risk Factors and Their Relation to Future Surgery for Valvular Heart Disease or Ascending Aortic Disease: A Case-Referent Study. *J Am Heart Assoc*. 2017;6. doi: 10.1161/JAHA.116.005133
