# Supplementary material for: Genomic and transcriptomic analyses of aortic stenosis enhance therapeutic target discovery and disease prediction
Source: Nat Genet. 2025 Dec 19;58(1):57–66. doi: 10.1038/s41588-025-02417-6 (PMC12807872; doi:10.1038/s41588-025-02417-6)
Supplement: Supplementary file 2 — Reporting Summary [file 41588_2025_2417_MOESM2_ESM.pdf]

nature portfolio

Corresponding author(s): George Thanassoulis
Pradeep Natarajan

Last updated by author(s): Oct 20, 2025

Reporting Summary

Nature Portfolio wishes to improve the reproducibility of the work that we publish. This form provides structure for consistency and transparency in reporting. For further information on Nature Portfolio policies, see our Editorial Policies and the Editorial Policy Checklist.

Statistics

For all statistical analyses, confirm that the following items are present in the figure legend, table legend, main text, or Methods section.

n/a	Confirmed	
☐	☒	The exact sample size (*n*) for each experimental group/condition, given as a discrete number and unit of measurement
☐	☒	A statement on whether measurements were taken from distinct samples or whether the same sample was measured repeatedly
☐	☒	The statistical test(s) used AND whether they are one- or two-sided<br>*Only common tests should be described solely by name; describe more complex techniques in the Methods section.*
☐	☒	A description of all covariates tested
☐	☒	A description of any assumptions or corrections, such as tests of normality and adjustment for multiple comparisons
☐	☒	A full description of the statistical parameters including central tendency (e.g. means) or other basic estimates (e.g. regression coefficient) AND variation (e.g. standard deviation) or associated estimates of uncertainty (e.g. confidence intervals)
☐	☒	For null hypothesis testing, the test statistic (e.g. *F*, *t*, *r*) with confidence intervals, effect sizes, degrees of freedom and *P* value noted<br>*Give P values as exact values whenever suitable.*
☒	☐	For Bayesian analysis, information on the choice of priors and Markov chain Monte Carlo settings
☒	☐	For hierarchical and complex designs, identification of the appropriate level for tests and full reporting of outcomes
☐	☒	Estimates of effect sizes (e.g. Cohen's *d*, Pearson's *r*), indicating how they were calculated

Our web collection on statistics for biologists contains articles on many of the points above.

Software and code

Policy information about availability of computer code

Data collection	All genetic and/or human aortic valve data was collected after informed consent according to pre-specified operating protocols for each biobank or study, which are described in detail in the Supplemental Methods.
Data analysis	Publicly available software was used to perform the analyses. Software packages, versions, and associated URLs are as follows: R statistical software 4.1 https://www.r-project.org/. LDSC 1.0.1 https://github.com/bulik/ldsc. liftOver v1.04.00 https://liftover.broadinstitute.org/. GWAMA v2.2.2 https://genomics.ut.ee/en/tools. PredictDB v7 https://predictdb.org/. MetaXcan v0.7.4 https://github.com/hakyimlab/MetaXcan. COLOC v3.2.1 https://chr1swallace.github.io/coloc/. QTLtools v1.1 https://qtltools.github.io/qtltools/. LocusCompareR v1.0.0 https://github.com/boxiangliu/locuscomparer. DEPICT v1 https://github.com/perslab/depict. apcluster v1.4.11 https://github.com/UBod/apcluster. Enrichr https://maayanlab.cloud/Enrichr/. LDPred2 was implemented through the R package bigsnpr v1.12.2 https://privefl.github.io/bigsnpr/ and bigstatsr v1.6.1 https://privefl.github.io/bigstatsr/ . PRS-CS (June 4, 2021) https://github.com/getian107/PRScs.

For manuscripts utilizing custom algorithms or software that are central to the research but not yet described in published literature, software must be made available to editors and reviewers. We strongly encourage code deposition in a community repository (e.g. GitHub). See the Nature Portfolio guidelines for submitting code & software for further information.

Data

Policy information about availability of data

All manuscripts must include a data availability statement. This statement should provide the following information, where applicable:

- Accession codes, unique identifiers, or web links for publicly available datasets
- A description of any restrictions on data availability
- For clinical datasets or third party data, please ensure that the statement adheres to our policy

Summary statistics from our multi-ancestry genome-wide association study, and all stratified analyses (by ancestry and sex) are available in the CVD Knowledge Portal (https://cvd.hugeamp.org/dinspector.html?dataset=Small2025_AorticStenosis). PRSs for aortic stenosis are available in the Polygenic Score Catalog (PGS005252; https://www.pgscatalog.org/). Researchers can apply for data from contributing biobanks following their data application procedures. We used publicly available human eQTL data from GTEx v8 https://gtexportal.org/home/.

Research involving human participants, their data, or biological material

Policy information about studies with human participants or human data. See also policy information about sex, gender (identity/presentation), and sexual orientation and race, ethnicity and racism.

Reporting on sex and gender	Biological sex information was determined using genotypes. Sex is used as a covariate in all genetic association experiments and sex-stratified analyses.
Reporting on race, ethnicity, or other socially relevant groupings	Genetic ancestry was determined in a study-specific manner. Details on ancestry determination is available in the published other socially relevant literature for all participating cohorts, which are detailed in our supplementary materials. Genetic association studies were groupings performed in ancestry stratified populations and then meta-analyzed.
Population characteristics	Population characteristics, including age and sex, are described by study in Supplemental Table 1.
Recruitment	Recruitment is study specific but mainly occurred as part of routine health care encounters after informed consent. As such, there may be selection bias in that many studies are enriched for individuals with disease (as occurs in a hospital setting). Further, diagnosis of aortic stenosis was determined by clinical coding and not routine screening. This means that there may be cases of aortic stenosis in the control population that were not properly coded or diagnosed. However we estimate that the number of missed cases is very small compared to the large (almost 3 million) number of controls and if anything would bias genetic discovery toward the null.
Ethics oversight	This work represents a discovery genome-wide association study of a pre-specified outcome (aortic stenosis). No treatment effects were studied; hence, there is no need for blinding in this work.

Note that full information on the approval of the study protocol must also be provided in the manuscript.

Field-specific reporting

Please select the one below that is the best fit for your research. If you are not sure, read the appropriate sections before making your selection.

☒ Life sciences ☐ Behavioural & social sciences ☐ Ecological, evolutionary & environmental sciences

For a reference copy of the document with all sections, see nature.com/documents/nr-reporting-summary-flat.pdf

Life sciences study design

All studies must disclose on these points even when the disclosure is negative.

Sample size	Sample size was determined based on study specific biobank recruitment. All available samples were analyzed.
Data exclusions	Data exclusions were specified in the phenotype definition, which is available in the supplement.
Replication	We implemented several measures to ensure the reproducibility of our experimental findings. All experiments were performed using biological and/or technical replicates, as appropriate, and each experiment was repeated independently using at least 5 donors of aortic valves/valve interstitial cells to confirm consistency. Detailed protocols, standardized reagents, and calibrated equipment were used throughout the study to minimize variability. All key findings were successfully replicated across independent experiments. There were no instances where findings could not be reproduced. Minor variability observed across replicates fell within expected experimental ranges and did not affect the overall conclusions. Therefore, we confirm that all attempts at replication were successful, supporting the robustness and reliability of our results.
Randomization	This work represents a discovery genome-wide association study of a pre-specified outcome (aortic stenosis). No treatment effects were studied; hence, there is no need for randomization in this work.
Blinding	This work represents a discovery genome-wide association study of a pre-specified outcome (aortic stenosis). No treatment effects were

Blinding	studied; hence, there is no need for blinding in this work.

Reporting for specific materials, systems and methods

We require information from authors about some types of materials, experimental systems and methods used in many studies. Here, indicate whether each material, system or method listed is relevant to your study. If you are not sure if a list item applies to your research, read the appropriate section before selecting a response.

Materials & experimental systems

n/a	Involved in the study
☒	☐ Antibodies
☒	☐ Eukaryotic cell lines
☒	☐ Palaeontology and archaeology
☒	☐ Animals and other organisms
☐	☒ Clinical data
☒	☐ Dual use research of concern
☒	☐ Plants

Methods

n/a	Involved in the study
☒	☐ ChIP-seq
☒	☐ Flow cytometry
☒	☐ MRI-based neuroimaging

Clinical data

Policy information about clinical studies

All manuscripts should comply with the ICMJE guidelines for publication of clinical research and a completed CONSORT checklist must be included with all submissions.

Clinical trial registration	NA
Study protocol	NA
Data collection	Data collection is study specific and described in the supplement.
Outcomes	Outcomes collection is study specific and described in the supplement.

Plants

Seed stocks	*Report on the source of all seed stocks or other plant material used. If applicable, state the seed stock centre and catalogue number. If plant specimens were collected from the field, describe the collection location, date and sampling procedures.*
Novel plant genotypes	*Describe the methods by which all novel plant genotypes were produced. This includes those generated by transgenic approaches, gene editing, chemical/radiation-based mutagenesis and hybridization. For transgenic lines, describe the transformation method, the number of independent lines analyzed and the generation upon which experiments were performed. For gene-edited lines, describe the editor used, the endogenous sequence targeted for editing, the targeting guide RNA sequence (if applicable) and how the editor was applied.*
Authentication	*Describe any authentication procedures for each seed stock used or novel genotype generated. Describe any experiments used to assess the effect of a mutation and, where applicable, how potential secondary effects (e.g. second site T-DNA insertions, mosiacism, off-target gene editing) were examined.*
